# Effect of Exercise Intensity on Spontaneous Physical Activity Energy Expenditure in Overweight Boys: A Crossover Study

**DOI:** 10.1371/journal.pone.0147141

**Published:** 2016-01-15

**Authors:** Vitor Barreto Paravidino, Mauro Felippe Felix Mediano, Daniel J. Hoffman, Rosely Sichieri

**Affiliations:** 1 Department of Epidemiology, Institute of Social Medicine, State University of Rio de Janeiro, Rio de Janeiro, Brazil; 2 Department of Physical Education and Sports, Naval School—Brazilian Navy, Rio de Janeiro, Rio de Janeiro, Brazil; 3 Evandro Chagas National Institute of Infectious Disease, Oswaldo Cruz Foundation, Rio de Janeiro, Rio de Janeiro, Brazil; 4 Department of Nutritional Sciences, Rutgers University, New Brunswick, New Jersey, United States of America; University of Tolima, COLOMBIA

## Abstract

**Objective:**

Evaluate the effect of different exercise intensities on spontaneous physical activity energy expenditure in overweight adolescents.

**Methods:**

A crossover study was developed with a control session, followed by moderate and vigorous exercise sessions, with six days of monitoring each. Twenty-four adolescents, 11–13 years old, male and overweight were selected. Spontaneous physical activity energy expenditure was assessed by accelerometers. Linear mixed effects models were used to evaluate the differences per session across time.

**Results:**

Energy expenditure during the 1st hour was different between all three sessions, with averages of 82, 286 and 343 kcal to the control, moderate and vigorous sessions, respectively (p <0.001). The same pattern of difference in energy expenditure between the sessions remained after 24 hours (704 vs 970 vs 1056 kcal, p <0.001). However, energy expenditure during the six days indicates compensation from second to the sixth day, although small differences remained at the end of the 6-day period (5102 vs 5193 vs 5271 kcal, p <0.001).

**Conclusions:**

A single aerobic session seems to modify the spontaneous physical activities in overweight adolescents but still keeping the vigorous session with higher total energy expenditure during the follow-up period. Despite the observed compensatory effect, the greater energy expenditure observed in both moderate and vigorous exercise sessions indicates that physical activity should be recommended to promote an increased energy expenditure in adolescents.

**Trial Registration:**

ClinicalTrials.gov NCT 02272088

## Introduction

Obesity is an important current public health problem, affecting approximately 35 to 40% of adults and 15 to 25% of children and adolescents worldwide [1]. The most important determinants of obesity in children and adolescents are inadequate eating habits associated with low levels of physical activity and sedentary lifestyle [2–4]. Several government agencies and professional organizations have recommended exercise for weight control [5–7]. For children and adolescents, at least 60 minutes of moderate to vigorous physical exercise is recommended daily for the maintenance of health and for the prevention of obesity and other diseases [8,9]. However, the reviews published in recent years regarding the effect of physical activity on weight control have showed conflicting results [10–12]. Thus, it is important and necessary to improve the understanding of how exercise regimens contribute to changes in daily energy expenditure, the aim of this study.

Intervention studies focused on physical activity reported that such interventions did not promote changes on BMI in children and adolescents [10,11]. Still, others have shown that physical exercise programs are able to enhance physical fitness and change body composition and several other risk factors for cardiovascular disease [12]. This controversy may be related to the fact that some studies have reported a lower energy deficit induced by exercise sessions when compared to the energy expenditure theoretically predicted [13,14], which can be explained by a possible compensatory effect on physical activities throughout the day [15,16]. The first researchers to address this phenomenon, Epstein & Wing [17] observed that patients undergoing exercise sessions had less weight loss than expected, suggesting that those who exercised modified their pattern of physical activity during the rest of the day. In other words, participants moved less to maintain an overall stable level of physical activity or energy expenditure over time, being described later as the activitystat hypothesis [18]. Although studies about this issue in children and adolescents have already been published [19,20], the results about the existence of compensatory mechanism are still conflicting [21]. Furthermore, most studies involved adults and the comparison of different exercise intensities has not been explored enough [21].

Despite the fact that there is a consensus in the literature about the recommendation of physical activity for children and adolescents, the intensity of exercise that should be recommended for weight loss remains unclear and the compensatory effect promoted by different exercise intensities on subsequent physical activities needs to be tested. Therefore, the objective of the present study was to determine the effect of exercise intensity on spontaneous physical activity energy expenditure among overweight adolescents.

## Methods

### Study design and sample

An experimental crossover study was conducted during the school year of 2014 in a public school in Niterói, Rio de Janeiro, Brazil. Initially, all children enrolled in the 6th and 7th grade were invited to undergo anthropometric measurements to determine their body mass index (BMI). Only those adolescents classified as overweight according to World Health Organization parameters for age and sex [22] were included in the study. Adolescents who reported any cardiovascular diseases, musculoskeletal injuries or any other factors that preclude the realization of the exercise protocols, measured by using the PAR-Q questionnaire (Physical Activity Readiness Questionnaire), were excluded from the study [23].

The written informed consent was obtained from all participants before the beginning of the study. This study was approved by the Research Ethics Committee of the Institute of Social Medicine of the State University of Rio de Janeiro.

The protocol for this trial and supporting TREND statement checklist are available as supporting information

### Intervention

The intervention protocol consisted of three sessions (control, moderate and vigorous exercise) for each adolescent, with intervals of at least 21 days between sessions and the participants blinded to the order of the sessions. Each child participated in all three of the intervention protocols to generate data for three separate intervention sessions. The determination of the intensity of each exercise session was based on the percentage of maximum heart rate achieved in a field test (Shuttle Run Test) carried out the week before the intervention protocol.

The control session consisted of the placement of accelerometers to assess energy expenditure and physical activity for a period of six days, without any specific physical exercise protocol. In the other sessions (moderate and vigorous), following the placement of accelerometers as described above, specific protocols of physical training with different intensities were performed on the first day of monitoring. No specific counseling to change the daily routine of adolescents during this week was provided.

Exercise sessions, lasting 60 minutes, were divided into three phases: warm-up (2 minutes and 30 seconds), training (55 minutes) and cool-down (2 minutes and 30 seconds). The exercise protocol was different between the experimental sessions (moderate and vigorous) only in the training phase, with similar warm-up and cool-down phases in both sessions. During the warm-up, subjects were instructed to start walking at a low-intensity, and then increase their pace until they reached values close to 64% of maximum heart rate. In the cool-down phase, adolescents were instructed to gradually reduce their pace in order to reach heart rate values close to those found at rest.

The main part of the moderate session (MS) consisted of 4 sets of 10 minutes walking at moderate intensity (64% to 76% of maximum heart rate), interspersed with 5 minutes of light walking (below 64% of maximum heart rate) for recovery between sets. The main part of vigorous session (VS) consisted on 4 sets of 10 minutes running at vigorous intensity (77% to 95% of maximum heart rate), interspersed with 5 minutes of light walking (below 64% of maximum heart rate) for the recovery between sets.

During the two experimental sessions, the adolescents were supervised by a trained exercise physiologist to ensure that they performed the intervention protocol properly. The target heart rate was monitored using heart rate monitors by the same professionals who supervised the training sessions.

### Measurement procedures

#### Anthropometric measurements

Body weight was measured using a portable electronic scale (Tanita BC -558 Japan) with a capacity of 150 kg and precision of 50g, while wearing light clothes and no shoes. Height was measured using a portable stadiometer (Alturexata, Brazil) with an amplitude of 200 cm and variation of 0.1 cm. The classification of nutritional status was based on BMI (kg/m^2^) cutoff points recommended by the World Health Organization [22].

#### Shuttle run test

The shuttle run test was conducted on a 20 meter field where the participant runs forward and then returns at a speed imposed by beeps (recording test protocol) and is a validated and widely used test in studies with adolescents [24,25]. The test was completed with the participant's withdrawal or when the participant failed to reach the demarcation line two consecutive times, as recommended. At the end of the test, the participants performed a light walk for at least 3 minutes to recover.

Each participant used a heart rate monitor during the test and the maximum heart rate was recorded. Thus, the intensity of each exercise session (moderate and vigorous) was calculated, individually, according to the percentage of the maximum frequency established for moderate and vigorous intensity [26].

A medical team was on standby during the test to provide fast treatment in case of complications.

#### Energy expenditure measurements

Energy expenditure for physical activity was measured using triaxial accelerometers (Actical—Phillips—Respironics, Oregon, USA) [27] attached to an elastic belt positioned on the right hip (anterior iliac crest). The accelerometer was placed on the same day of the week and time of day for all sessions and removed after six days. Participants were instructed to not remove the device during this period, except while bathing and during water activities.

Based on counts values, kcal was estimated according to Actical software parameters. The criteria used to define the non-use time of accelerometers was 60 consecutive minutes of zero counts or 60 consecutive minutes of zero energy expenditure and a non-valid day was defined as those days which non-use time was greater than 14 hours. Therefore, individuals were excluded from the analysis of the day if they failed to provide a minimum of 600 min (10 h) of valid data [28].

#### Sample size

Sample size was calculated based on a mean difference in daily energy expenditure between sessions of 110 kcal [29], with a coefficient of variation equal to 1 (standard deviation of 110 kcal) [30]. The sample size required for the study, with an α of 0.05 (two-sided) and β of 0.10 and estimating a refusal rate of 20%, was 27 adolescents [31]. Only 24 adolescents participated in the study, however sample size calculation did not take into account repeated measures, which increases the power of the study [32].

### Data Analysis

The descriptive analyses were calculated the mean and standard deviations for continuous and percentages for categorical variables. The Kurtosis and Skewness test using Stata 13.0 software was performed to assess the normality of the data.

Energy expenditure associated with physical activity was assessed in the 1st hour of accelerometer usage, which corresponds to the period of the exercise protocol for moderate and vigorous sessions, and over the course of six days. Cumulative total energy expenditure was estimated from accelerometer data for 24, 48, 72, 96, 120 and 144 hours. Differences between sessions for energy expenditure in the first hour were performed by using a one-way ANOVA followed by a post-hoc Scheffé test. Differences in daily and accumulated energy expenditure between exercise sessions was performed using linear mixed models, which takes into account the correlations between repeated measures over time. The model incorporated a quadratic term (time X time) for those analyses which demonstrated a non-linear change (p<0.05). We tested the covariance structure of the models and the unstructured covariance matrix appeared to be the most adequate for these data. All of the analysis was performed in SAS 9.3 (Statistical Analysis System, USA) and STATA 13.0 software. Statistical significance was set at p<0.05 for all analyses.

## Results

**Fig 1:**
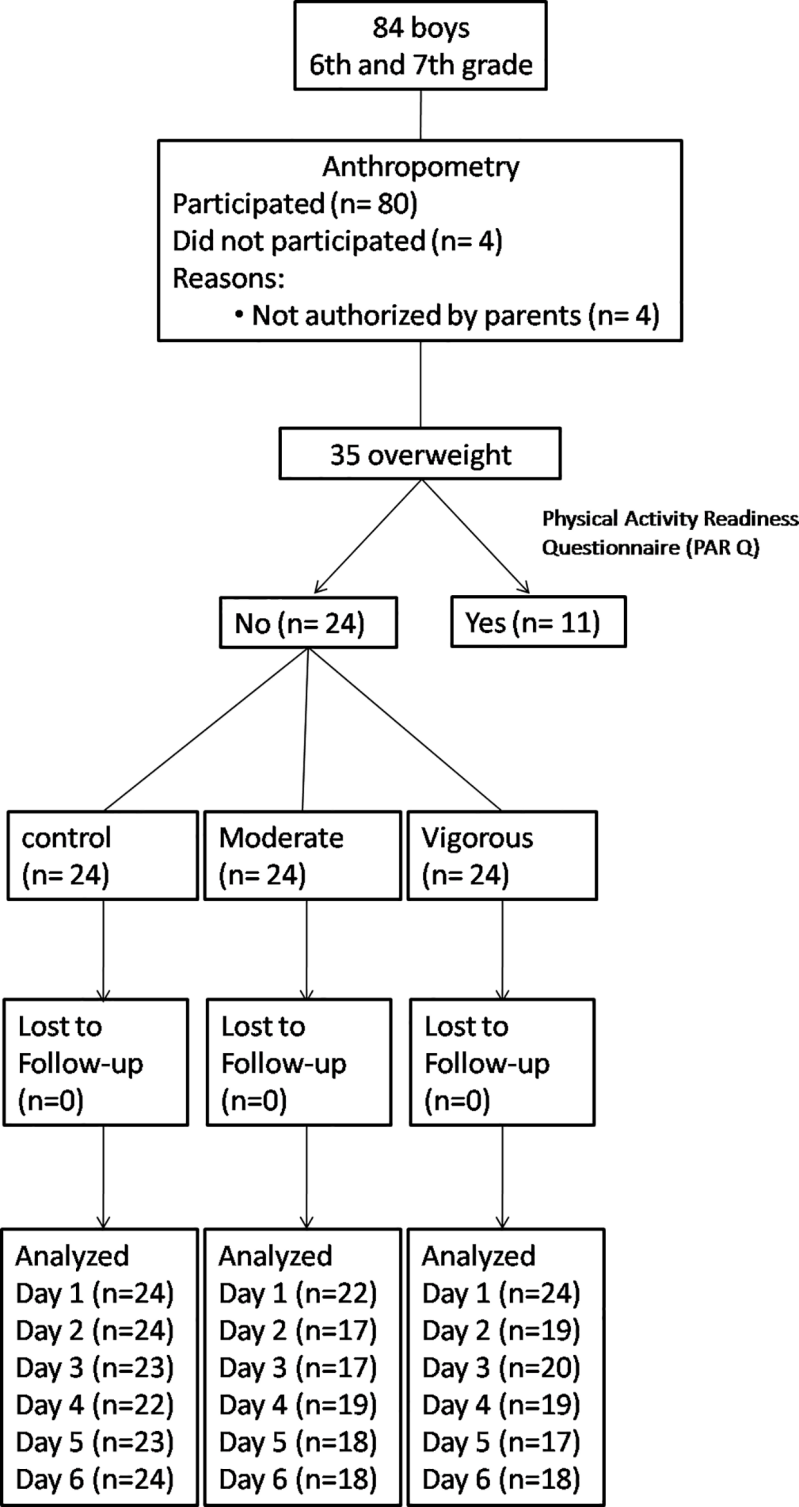
Flow diagram of participants through the study.

Of the 84 children enrolled in 6th and 7th grade, 24 met the criteria for inclusion determined in the study and were selected for the research protocol (Fig 1). No adverse events were observed during training sessions.

Physical characteristics and baseline data for all participants are summarized in Table 1.

**Table 1:** Baseline characteristics of participants included in the study.

Variables (n = 24)	Mean (SD*)
Age (years)	12.6 (0.95)
Stature (cm)	158.5 (10.13)
Weight (kg)	60.9 (11.89)
BMI (kg/ m2)	24.0 (2.57)
HR máx s*huttle test* (bpm)	191.9 (8.24)

* standard deviation

Non-valid days for energy expenditure were observed in two subjects in the control session (one on the third day and one on the fourth day), 14 in the moderate session (two in the first six in the second, three in the third day, two in the fifth and one in sixth), and 10 in the vigorous session (five in the second, one on the third day, one on the fourth and three in the fifth), with these values (> 14 hours of zeros counts) excluded from the analysis.

Energy expenditure during the first hour following the intervention (Table 2) was significantly greater in the vigorous and moderate sessions compared to the control session (p <0.001) and greater in the vigorous compared to the moderate session (p = 0.04).

**Table 2:** Energy expenditure (EE, kcal) in physical activity in the first hour of registration.

Sessions	EE hour1	p-value*	Post-hoc	*p-value* ^*#*^
Control	82 (7)		Control vs. Moderate	< 0.001
Moderate	286 (16)	< 0.001	Control vs. Vigorous	< 0.001
Vigorous	343 (21)		Moderate vs. Vigorous	0.04

* Analysis of variance (one-way ANOVA)

^#^ *Post hoc* Scheffé

**Fig 2:**
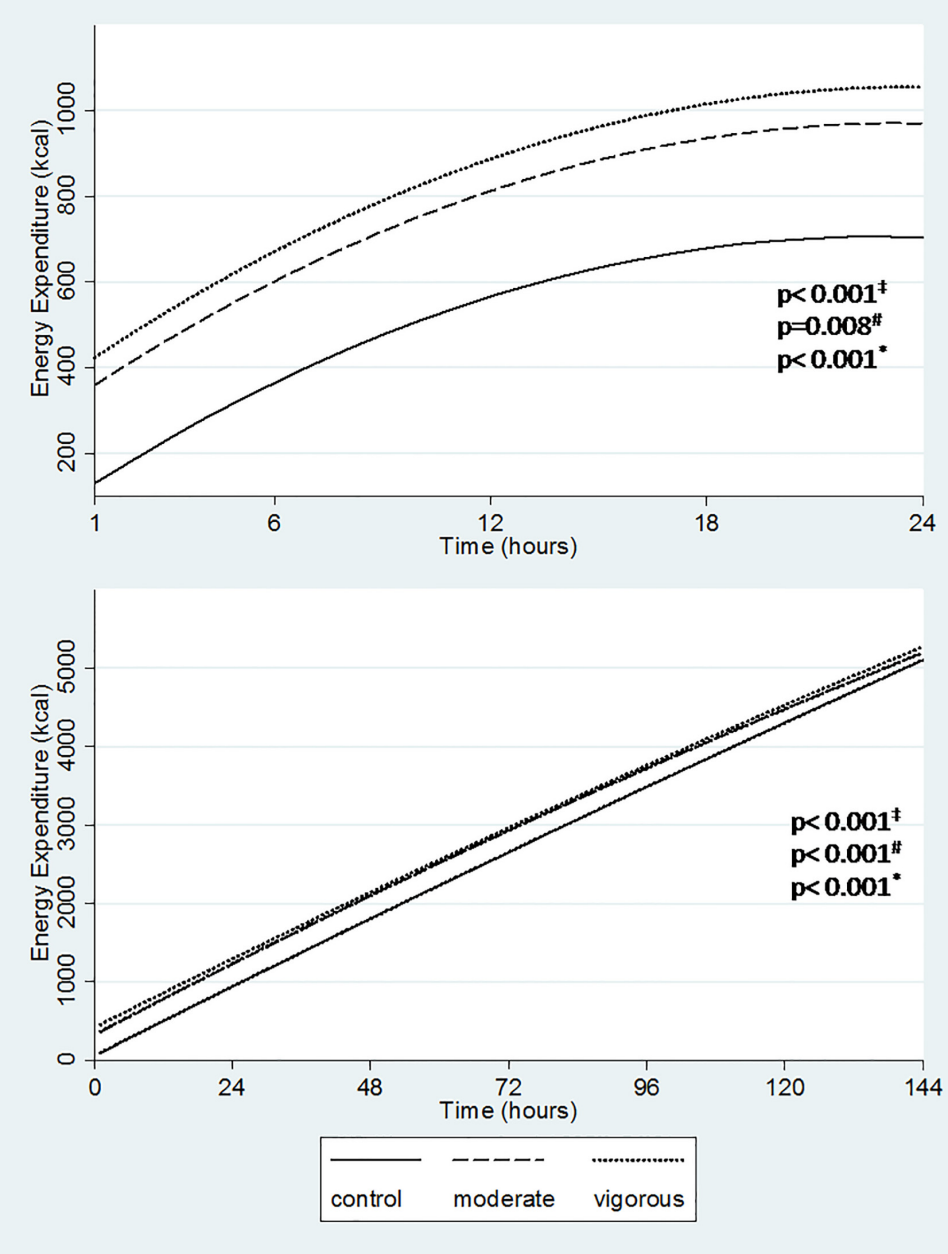
Estimated mean values of the cumulative energy expenditure in 24 hours (A) and 144 hours (B) monitoring. ‡ moderate vs. control; # moderate vs. vigorous; * vigorous vs. control.

For the 24 hours following each intervention (Table 3), energy expenditure was greater in the vigorous compared to the moderate and control sessions (1056 vs. 970 and 704 kcal, respectively, p = 0.008) and in the moderate compared to the control session (970 vs. 704 kcal, respectively, p <0.001) (Fig 2A). Cumulative total energy expenditure for the six days following each intervention was higher for the vigorous session compared to the moderate (5271 vs. 5193 kcal, p <0.001) and the control sessions (5271 vs. 5102 kcal, p <0.001). The cumulative total energy expenditure for the six days following the moderate was higher compared to that following the control session (5193 vs. 5102 kcal, p <0.001) (Fig 2B). Differences in cumulative total energy expenditure between control and exercise sessions diminished at the end of the six-day period (Table 3).

**Table 3:** Estimated means* and confidence intervals of energy expenditure with physical activities (kcal) within 24 hours and 144 hours of monitoring.

Sessions	24 hours*	95% CI	144 hours*	95% CI
Control	704	603–804	5102	4694–5511
Moderate	970	869–1071	5193	4783–5603
Vigorous	1056	955–1156	5271	4862–5680

*Linear mixed model with time, group, group*time, time * time, group*time*time.

**Fig 3:**
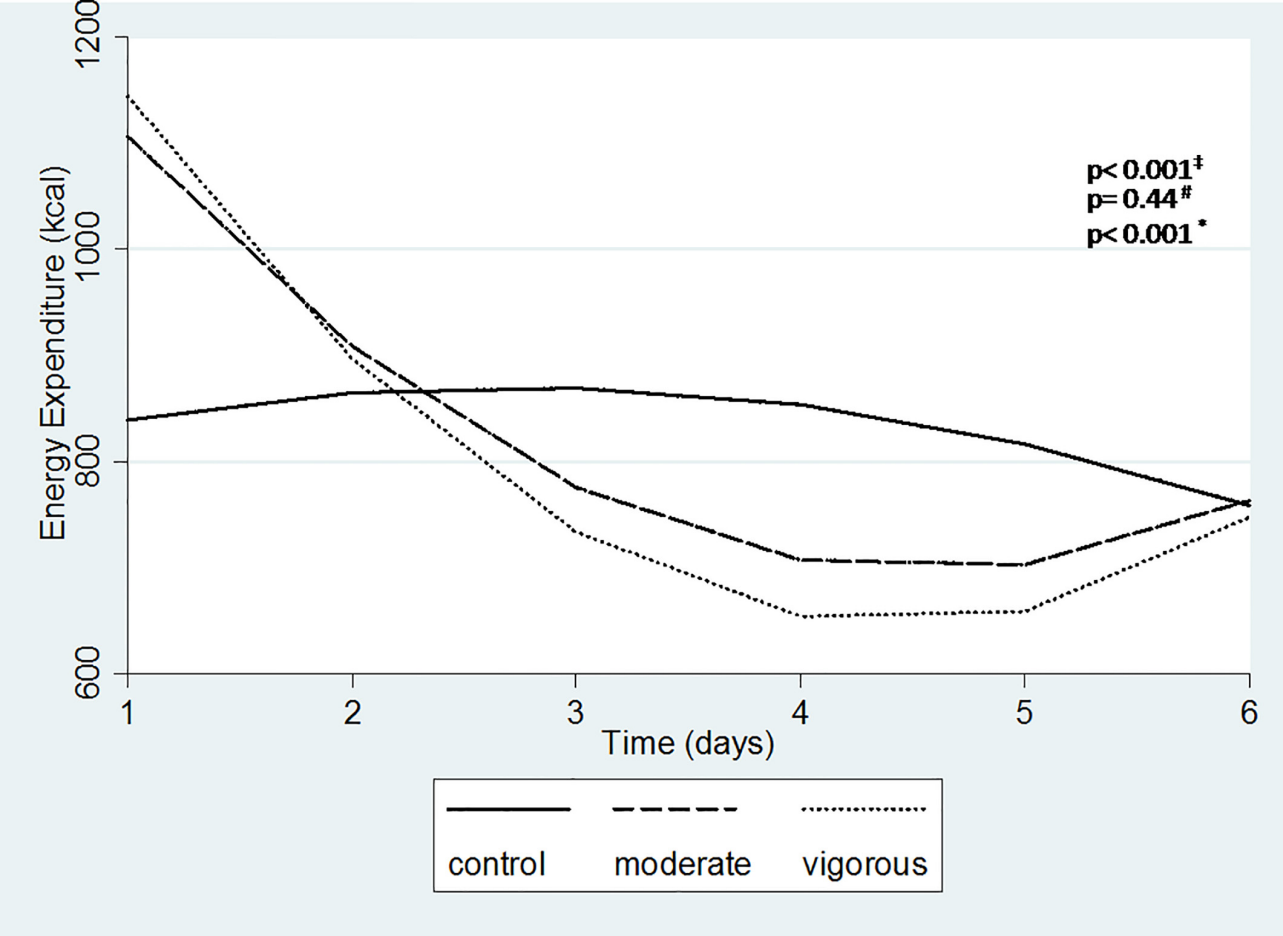
Estimated mean values of energy expenditure per day for 6 days monitoring. Linear mixed model with time, group * time, time*time, group * time * time, adjusted for EE for the 1st hour. ‡ moderate vs. control; # moderate vs. vigorous; * vigorous vs. control.

The daily energy expenditure (not accumulated) was significantly lower in the moderate and vigorous sessions compared to the control session from the second to the sixth day following the exercise session, with significant differences in time trajectories between control and exercise sessions (p <0.001) (Fig 3).

## Discussion

The main finding of the present study was a change in spontaneous physical activity energy expenditure in overweight adolescents after a single bout of exercise, both for moderate and vigorous exercise intensities. Rowland (1998) describes the hypothesis of 'activitystat' as a homeostatic mechanism, where a biological center would be responsible for the physical activity control according to a set point of energy expenditure. According to this theory, modification in physical activity levels, at any given time, would be offset by changing these levels at another time, in defense of an individual set point [18].

In agreement with our findings, other studies conducted among children and adolescents support this theory [33,34]. Frémeaux et al. (2011) evaluated the effect of a higher volume of physical activity practiced at school on the volume of physical activity performed outside the school among children aged 8–10 years and found that an increased volume of physical activity practiced at school induces a reduction in the spontaneous physical activities throughout the day, demonstrating the presence of the compensatory effect [33].

Similarly, Wilkin et al. (2006) found that daily physical activity levels were similar among children who performed low or high volumes of physical activity at school, concluding that daily physical activity levels are not only dependent of the environmental characteristics in which they were inserted [34] but also by physiological mechanisms that regulate physical activity levels, reinforcing the importance of biological control theory [18]. Also, in a recent systematic review, Gomersall et al. (2013) highlighted that compensatory effects of exercise in reducing physical activity levels could occur even for several days after a single exercise bout [35], corroborating our results that reduction of physical activity energy expenditure in both exercise sessions was observed only after two days of monitoring and lasted until the sixth day.

Several intervention studies failed to demonstrate an effect of physical activity on weight loss among children and adolescents [11,36]. In a meta-analysis, Harris et al. (2009) observed no impact of school-based physical activity interventions on BMI of children and adolescents [36]. In addition, a systematic review conducted by Dobbins et al. (2013) did not observe an impact of physical activity programs performed at school on BMI in children and adolescents 6–18 years old [11]. Thus, the difficulty of obtaining weight loss with interventions based solely on physical exercise may be related to a lower energy deficit induced by exercise when compared to the theoretically predicted energy expenditure due to the compensatory effect.

However, in this study, cumulative total energy expenditure during the six days of follow-up was higher for vigorous and moderate sessions in comparison with control session. More important, the compensatory effect observed in exercise sessions was insufficient to equalize the cumulative energy expenditure between control and exercise sessions. Thus, even with compensatory effect, vigorous or moderate exercise should be encouraged among adolescents to achieve a negative energy balance and thus promote weight loss.

Although the results of the present study support the activitystat hypothesis, other studies report results counter to this theory [37–39]. Wickel & Eisenmann (2007) compared the amount of physical activity performed on days with and without sports practice. It was found that a sports practice was associated with a greater daily volume of moderate-to-vigorous physical activity (30 minutes) and a reduced amount of sedentary activities (40 minutes) and suggested that that sports practice does not induce the compensatory response in spontaneous physical activity. [38]. Likewise, Dale et al. (2000) observed that physical activity restriction during school time was not compensated by increasing physical activity in the period spent out of school [39]. The differences observed between these studies’ results could be explained by differences in exercise protocols, especially the type of exercise, frequency, duration and intensity of exercise sessions.

In the present study, there was no influence of exercise intensity on energy expenditure with physical activities during the six days of monitoring, with a similar compensatory effect observed for the second to the sixth day in both moderate and vigorous sessions. However, other studies have reported different results [19,20]. Kriemler et al. (1999) observed in obese adolescents, using heart rate monitor, that energy expenditure with physical activities was reduced by 3% on the day of moderate exercise session and 6% on the day of vigorous session and, in the day after, moderate exercise caused an increase on energy expenditure while vigorous exercise caused a reduction [19]. Thivel et al. [20] published a study about the effect of exercise intensity on energy expenditure and found that obese adolescents demonstrated a compensatory response to high-intensity exercises (above 70% of VO2 max), therefore reducing the energy expenditure of subsequent physical activities. Thus, due to the conflicting results reported in the literature, further studies are needed to formulate a solid base on the effects of different exercise intensities on energy expenditure with spontaneous physical activity, the mechanisms involved and their determinant factors.

The present study has some limitations that require discussion to better understand the context in which our results are presented. First, due to the inclusion of overweight adolescents and their low exercise tolerance, vigorous exercise was performed near to the lower limit of the target heart rate. Thus, differences between exercise intensities could have not been sufficient to promote differences for compensatory effect between the exercise sessions. On the other hand, there was significant difference to energy expenditure during moderate to vigorous exercise sessions, which indicates that the intensity of exercise performed in the two sessions was really different. Second, the practice of the exercise sessions during the same period of the year does not guarantee stability in temperature and humidity, and may therefore influence the performance of the participants during the exercise protocol. Third, the present study evaluated the effect of a single exercise session on energy expenditure with physical activities over 6 days, preventing the assessment of the compensatory effect after multiple sessions of physical exercise. Fourth, even though the shuttle run test is not a gold standard to evaluate the maximal exercise capacity, it is a validated and widely used test in studies with adolescents. Finally, the present study used only overweight boys, making it difficult to extrapolate our results to other populations.

## Conclusion

In conclusion, based on the results of our study, we found that a single aerobic exercise session modifies the level of spontaneous physical activity among overweight adolescents. In agreement with the physical exercise compensatory mechanism, the increase in physical activity at one time was offset by reducing the physical activity pattern at another time, in order to maintain a stable level over time. However, despite the observed compensatory effect, higher six-day cumulative energy expenditure was observed for exercise sessions in comparison with control. Accordingly, the practice of moderate to vigorous intensity physical exercise should be encouraged for overweight adolescents in order to promote a negative energy balance and facilitate weight loss.

Future studies are needed to investigate the energy expenditure compensation in obese and non-obese individuals, the effect of different exercise intensities and a greater number of training sessions.

## Supporting Information

S1 TREND Checklist Trendstatement trend checklist (doc). (DOCX)

S1 Protocol Original protocol (doc). (DOC)

S2 Protocol Translated protocol (doc). (DOC)

## References

[1] Ng M, Fleming T, Robinson M, Thomson B, Graetz N, Margono C, et al Global, regional, and national prevalence of overweight and obesity in children and adults during 1980–2013: A systematic analysis for the Global Burden of Disease Study 2013. Lancet. 2014; 384: 766–781. 10.1016/S0140-6736(14)60460-8 24880830 PMC4624264

[2] World Health Organization. WHO Global strategy on diet, physical activity and health France: World Health Organization, 2009.

[3] Ludwig DS, Peterson KE, Gortmaker SL. Relation between consumption of sugar-sweetened drinks and childhood obesity: a prospective, observational analysis. Lancet. 2001; 357: 505–508. 11229668 10.1016/S0140-6736(00)04041-1

[4] Sichieri R. Consumo alimentar no Brasil e o desafio da alimentação saudável. ComCiência. 2013; 145.

[5] Saris WH, Blair SN, van Baak MA et al How much physical activity is enough to prevent unhealthy weight gain? Outcome of the IASO 1st Stock Conference and consensus statement. Obes Ver. 2003; 4: 101–114. 10.1046/j.1467-789x.2003.00101.x 12760445

[6] Institute of Medicine, Panel on Macronutrients, Standing Committee on the Scientific Evaluation of Dietary Reference Intakes. Dietary Reference Intakes for Energy, Carbohydrate, Fiber, Fat, Fatty Acids, Cholesterol, Protein, and Amino Acids. Natl Academy Press: Washington DC; 2005.

[7] Donnelly JE, Blair SN, Jakicic JM et al American College of Sports Medicine Position Stand. Appropriate physical activity intervention strategies for weight loss and prevention of weight regain for adults. Med Sci Sports Exerc. 2009; 41: 459–471. 10.1249/MSS.0b013e3181949333 19127177

[8] Tremblay A, Simoneau JA, Bouchard C. Impact of exercise intensity on body fatness and skeletal muscle metabolism. Metabolism. 1994; 43: 814–818. 8028502 10.1016/0026-0495(94)90259-3

[9] Landry BW, Driscoll SW. Physical activity in children and adolescents. PM R. 2012; 4: 826–832. 10.1016/j.pmrj.2012.09.585 23174545

[10] Harris KC, Kuramoto LK, Schulzer M, Retallack JE. Effect of school-based physical activity interventions on body mass index in children: a meta-analysis. CMAJ. 2009; 180:719–726. 10.1503/cmaj.080966 19332753 PMC2659836

[11] Dobbins M, Husson H, DeCorby K, LaRocca RL. School-based physical activity programs for promoting physical activity and fitness in children and adolescents aged 6 to 18. Cochrane Database Syst Rev. 2013; 2: CDOO7651. 10.1002/14651858.CD007651.pub2 PMC7197501 23450577

[12] Vasconcellos F, Seabra A, Katzmarzyk PT, Kraemer-Aguiar LG, Bouskela E, Farinatti P. Physical Activity in Overweight and Obese Adolescents: Systematic Review of the Effects on Physical Fitness Components and Cardiovascular Risk Factors. Sports Med. 2014; 44: 1139–1152. 10.1007/s40279-014-0193-7 24743931

[13] Church TS, Martin CK, Thompson AM et al Changes in weight, waist circumference and compensatory responses with different doses of exercise among sedentary, overweight postmenopausal women. Plos One. 2009; 4: e4515 10.1371/journal.pone.0004515 19223984 PMC2639700

[14] King NA, Caudwell P, Hopkins M et al Metabolic and behavioral compensatory responses to exercise interventions: barriers to weight loss. Obesity. 2007; 15: 1373–1383. 17557973 10.1038/oby.2007.164

[15] Westerterp K. Pattern and intensity of physical activity. Nature. 2001; 410: 539. 10.1038/35069142 11279482

[16] Manthou E, Gill JMR, Wright A, Malkova D. Behavioral compensatory adjustments to exercise training in overweight women. Med Sci Sports Exerc. 2010; 42: 1221–1228. 10.1249/MSS.0b013e3181c524b7 19997033

[17] Epstein LH, Wing RR. Aerobic exercise and weight. Addict Behav. 1980; 5:371–388. 7211534 10.1016/0306-4603(80)90011-8

[18] Rowland TW. The biological basis of physical activity. Med Sci Sports Exerc. 1998; 30: 392–399. 9526885 10.1097/00005768-199803000-00009

[19] Kriemler S, Hebestreit H, Mikami S, Bar-Or T, Ayub BV, Bar-Or O. Impact of a single exercise bout on energy expenditure and spontaneous physical activity of obese boys. Pediatr Res. 1999; 46: 40–44. 10400132 10.1203/00006450-199907000-00007

[20] Thivel D, Aucouturier J, Metz L, Morio B, Duche P. Is there spontaneous energy expenditure compensation in response to intensive exercise in obese youth? Pediatr Obes. 2014; 9:147–154. 10.1111/j.2047-6310.2013.00148.x 23447495

[21] Washburn RA, Lambourne K, Szabo AN, Herrmann SD, Honas JJ, Donnelly JE. Does increased prescribed exercise alter non-exercise physical activity/energy expenditure in healthy adults? A systematic review. Clin Obes. 2014; 4: 1–20. 10.1111/cob.12040 25425128 PMC5996763

[22] de Onis M, Onyango AW, Borghi E, Siyam A, Nishida C, Siekmann J. Development of a WHO growth reference for school-aged children and adolescents. Bull World Health Organ. 2007; 85: 660–667. 18026621 10.2471/BLT.07.043497 PMC2636412

[23] Shephard RJ, Cox MH, Simper K. An analysis of "PAR-Q" responses in an office population. Can J Public Health. 1981; 72: 37–40. 7225989

[24] Leger LA, Mercier D, Gadoury C, Lambert J. The multistage 20 metre shuttle run test for aerobic fitness. J Sports Sci. 1988; 6: 93–101. 3184250 10.1080/02640418808729800

[25] Veses AM, Martinez-Gomez D, Gomez-Martinez S, Vicente-Rodriguez G, Castillo R, Ortega FB, et al Physical fitness, overweight and the risk of eating disorders in adolescents. The AVENA and AFINOS studies. Pediatr Obes. 2014; 9:1–9. 10.1111/j.2047-6310.2012.00138.x 24449515

[26] American College of Sports Medicine. ACSM’ Guidelines for exercise testing and prescription 8th ed. Philadelphia: Lippincott Willians & Wilkins; 2009.

[27] Heil DP. Predicting activity energy expenditure using the Actical activity monitor. Res Q Exerc Sport. 2006; 77: 64–80. 16646354 10.1080/02701367.2006.10599333

[28] Wolff-Hughes DL, Bassett DR, Fitzhugh EC. Population-referenced percentiles for waist-worn accelerometer derived total activity counts in U.S. youth: 2003–2006 NHANES. Plos One. 2014; 9: 1–14. 10.1371/journal.pone.0115915 PMC4274159 25531290

[29] Wang YC, Gortmaker SL, Sobol AM, Kuntz KM. Estimating the energy gap among US children: a counterfactual approach. Pediatrics. 2006; 118: e1721–1733. 17142497 10.1542/peds.2006-0682

[30] van Belle G. Statistical rules of thumb 2nd ed. John Wiley and Sons: New York, 2008.

[31] Julious SA. Sample size for clinical trials Boca Raton: CRC Press; 2010.

[32] Fitzmaurice GM, Laird NM, Ware JH. Applied longitudinal analysis 2nd ed. New Jersey: Hoboken; 2011.

[33] Fremeaux AE, Mallam KM, Metcalf BS, Hosking J, Voss LD, Wilkin TJ. The impact of school-time activity on total physical activity: the activitystat hypothesis (EarlyBird 46). Int J Obes (Lond). 2011; 35:1277–1283. 21407175 10.1038/ijo.2011.52

[34] Wilkin TJ, Mallam KM, Metcalf BS, Jeffery AN, Voss LD. Variation in physical activity lies with the child, not his environment: evidence for an 'activitystat' in young children (EarlyBird 16). Int J Obes. 2006; 30: 1050–1055. 10.1038/sj.ijo.0803331 16801942

[35] Gomersall SR, Rowlands AV, English C, Maher C, Olds TS. The ActivityStat hypothesis: the concept, the evidence and the methodologies. Sports Med. 2013; 43:135–149. 10.1007/s40279-012-0008-7 23329607

[36] Harris KC, Kuramoto LK, Schulzer M, Retallack JE. Effect of school-based physical activity interventions on body mass index in children: a meta-analysis. CMAJ. 2009; 180: 719–726. 10.1503/cmaj.080966 19332753 PMC2659836

[37] Blaak EE, Westerterp KR, Bar-Or O, Wouters LJ, Saris WH. Total energy expenditure and spontaneous activity in relation to training in obese boys. Am J Clin Nutr. 1992; 55: 777–782. 1550058 10.1093/ajcn/55.4.777

[38] Wickel EE, Eisenmann JC. Contribution of youth sport to total daily physical activity among 6- to 12-yr-old boys. Med Sci Sports Exerc. 2007; 39:1493–1500. 17805079 10.1249/mss.0b013e318093f56a

[39] Dale D, Corbin CB, Dale KS. Restricting opportunities to be active during school time: do children compensate by increasing physical activity levels after school? Res Q Exerc Sport. 2000; 71: 240–248. 10999261 10.1080/02701367.2000.10608904

