# Supplementary material for: Effect of Exercise Intensity on Spontaneous Physical Activity Energy Expenditure in Overweight Boys: A Crossover Study
Source: PLoS One. 2016 Jan 15;11(1):e0147141. doi: 10.1371/journal.pone.0147141 (PMC4714875; doi:10.1371/journal.pone.0147141)
Supplement: S1 Protocol — (DOC) [file pone.0147141.s002.doc]

| 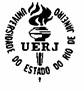 **No** | **UNIVERSIDADE DO ESTADO DO RIO DE JANEIRO**  **Instituto de Medicina Social**  Rua São Francisco Xavier, 524 / 7º andar / Blocos D e E - Maracanã  CEP: 20559.900 - Rio de Janeiro - RJ - BRASIL  TELS: 55-21-587-73 03/587-7540/587-7422/587-7572/284-8249  FAX: 55-21-264-1142  [http://www.ims.uerj.br](http://www.ims.uerj.br/)  ­­­­­ | 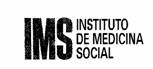 |
| --- | --- | --- |

**Projeto de pesquisa:** Efeito de diferentes intensidades do exercício físico no gasto energético diário com atividades físicas em meninos com excesso de peso

**Este é um projeto de mestrado, aprovado no exame de qualificação do dia 29 de julho de 2014.**

1. **Introdução**
   1. **Prevalência de excesso de peso, determinantes e consequências.**

As diversas mudanças associadas ao processo de modernização e industrialização sofridas pela sociedade nos últimos tempos têm refletido diretamente na vida dos indivíduos, resultando em mudanças importantes no padrão de atividade física e alimentação da população. Como consequência destas transformações, estudos epidemiológicos têm mostrado um rápido aumento na prevalência de sobrepeso e obesidade não somente em adultos, mas também em crianças e adolescentes (de Onis, et al., 2010; Swinburn, et al., 2011; Wang & Lim, 2012).

Estima-se que 1,46 bilhões de adultos em todo o mundo têm excesso de peso, e que destes, 502 milhões são obesos (Finucane, et al., 2011). Adicionalmente, dados da Organização Mundial da Saúde (OMS) mostram que aproximadamente 20% das crianças e adolescentes dos países ocidentais já apresentam quadro de excesso de peso (Saha, et al., 2011).

No Brasil, a partir da década de 70, os inquéritos alimentares começaram a avaliar a situação nutricional da população do país, e uma análise comparativa dessas pesquisas aponta declínio marcante da prevalência de desnutrição e um aumento expressivo de sobrepeso e obesidade (Batista Filho & Rissin, 2003).

A Pesquisa de Orçamentos Familiares (POF) 2008/2009 mostrou que o excesso de peso atinge 50,1% dos homens e 48,0% das mulheres e que a prevalência de obesidade alcançou 12,4% e 16,9% da população brasileira, respectivamente (**Gráfico 1**). Ao comparar o período 1974/1975 com 2008/2009, percebe-se que o excesso de peso entre os homens praticamente triplicou (de 18,5% para 50,1%) e que entre as mulheres foi quase duas vezes superior (de 28,7% para 48,0%). No mesmo período, a prevalência de obesidade aumenta em mais de quatro vezes para homens (de 2,8% para 12,4%) e em mais de duas vezes para mulheres (de 8,0% para 16,9%) (IBGE, 2010).

Os dados de 2013 do sistema de Vigilância de Fatores de Risco e Proteção para Doenças Crônicas por Inquérito Telefônico (VIGITEL) corroboram a tendência de aumento na prevalência de excesso de peso, principalmente no sexo masculino. No conjunto da população adulta das 27 cidades, a frequência de excesso de peso foi de 50,8%, sendo maior entre os homens (54,7%) do que entre as mulheres (47,4%) (Brasil, 2014).

Com relação às crianças e adolescentes, uma revisão sistemática de estudos de prevalência de excesso de peso realizados em diferentes regiões do Brasil aponta para valores variando entre 4% a 31% (Araújo, et al., 2012) e ainda com prevalências superiores a 37% nos estudos mais recentes (Kaufmann & Albernaz, 2013; Pardo, et al., 2013).

Esses dados se tornam extremamente relevantes para a saúde pública a partir do momento em que vários estudos têm mostrado que uma criança ou adolescente com excesso de peso apresenta maiores chances de apresentar esse quadro na vida adulta e, já nessa faixa etária, podem apresentar alterações psicossociais como, baixa autoestima, autoimagem negativa, transtornos alimentares e pior qualidade de vida (Sharma, 2006; Herman, et al., 2009; Juhola, et al., 2011; Reilly & Kelly, 2011), além de apresentar aumentar risco aumentado para desenvolvimento de comorbidades como, hipertensão arterial, dislipidemia, hiperinsulidemia, diabetes mellitus tipo 2, síndrome da apneia do sono, asma e complicações ortopédicas (Kiess, et al., 2003; Gidding, et al., 2004; Laska, et al., 2012; Raj, 2012).

Diferenças no estado nutricional podem ser decorrentes tanto da influência genética, do meio ambiente ou da interação entre ambos. Os fatores determinantes da obesidade não estão totalmente esclarecidos, entretanto a elevada prevalência de hábitos alimentares inadequados, com elevação da densidade calórica das refeições, da quantidade de alimentos consumidos e do aumento do consumo de bebidas adoçadas (Ludwig, et al., 2001; Sichieri, 2013) associados à inatividade física e à hábitos sedentários são considerados os principais contribuintes para a crescente ocorrência da obesidade e demais doenças crônicas não-transmissíveis.

A complexidade dos fatores associados a obesidade é esquematizada na **figura 1**, que tenta identificar alguns dos potenciais fatores determinantes e suas possíveis inter-relações em crianças (Monasta, et al., 2010). Os resultados desta revisão indicam que a amamentação pode ser um fator de proteção para obesidade futura enquanto que a obesidade na infância, rápido crescimento infantil, diabetes gestacional, tabagismo materno, poucas ou excesso de horas de sono, menos de 30 minutos de atividade física diária e consumo de bebidas adoçadas podem ser considerados importantes fatores de risco. Obesidade dos pais, crescimento intrauterino e peso ao nascer inadequados, tempo de televisão, insegurança alimentar e baixo nível socioeconômico também podem ser incluídos entre os fatores de risco embora seja difícil de estimar a magnitude do efeito.


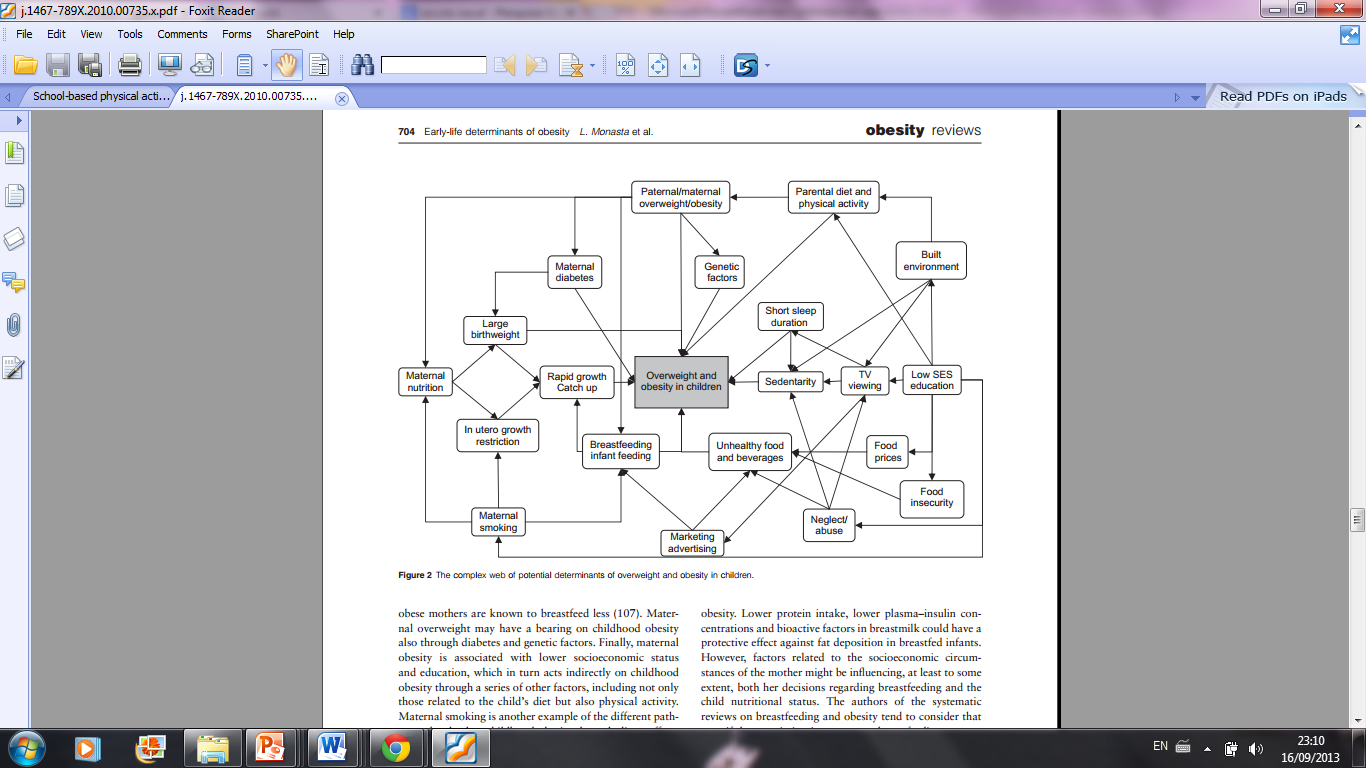


**Figura 1.** Potenciais determinantes do sobrepeso em crianças e suas possíveis inter-relações (retirado de Monasta et al., 2010).

A identificação dos potenciais determinantes e o melhor entendimento de suas relações podem orientar a adoção de políticas públicas que promovam o enfrentamento do problema da obesidade na população.

- 1. **O exercício físico em programas de prevenção de obesidade**

A inatividade física é considerada pela OMS como um dos principais fatores de risco para o desenvolvimento de diversas doenças crônicas degenerativas como as doenças cardiovasculares, diabetes, osteoporose e alguns tipos de câncer (World Health Organization, 2010). A manutenção de uma vida ativa e a redução de atividades sedentárias estão associadas à baixos índices da adiposidade corporal, melhora da saúde cardiovascular, óssea e muscular, redução da ansiedade e depressão, entre outros (Janssen, et al., 2010; Ravussin, et al., 1988; Zurlo, et al., 1992; Weinsier, et al., 1998; Pate, et al., 2013).

Sendo assim, muitas organizações e sociedades científicas publicaram recomendações para a promoção da atividade física e a utilização do exercício físico como estratégia no tratamento da obesidade, diabetes mellitus e para a prevenção de doenças cardiovasculares (Jakicic, et al., 2001; Bauman, et al., 2005; Warburton, et al., 2007). Para adultos, recomenda-se a prática semanal de pelo menos 150 minutos de exercícios aeróbios leves ou moderados ou 75 minutos de exercícios aeróbios intensos complementados por 3 sessões semanais de exercícios resistidos (World Health Organization, 2010; Garber, et al., 2011). Com relação às crianças e adolescentes, posicionamentos recentes no Brasil e no exterior têm recomendado a prática diária de exercícios físicos, com duração mínima de 60 minutos, de intensidade moderada a vigorosa, tanto para a manutenção da saúde quanto para a prevenção da obesidade e outras doenças (Tremblay, et al., 1994; Landry & Driscoll, 2012).

Hallal et al., (2012) apresentaram os níveis mundiais de atividade física com dados obtidos de adultos (15 anos ou mais) de 122 países e de adolescentes (13 a 15 anos) de 105 países. Os resultados demonstraram que 31,1% dos adultos em todo o mundo são fisicamente inativos, com proporções variando entre 17% no sul da Ásia a 43% nas Américas e no Mediterrâneo Oriental. Foi observado ainda que a inatividade física aumenta com a idade, é maior entre as mulheres e mais elevada em países de alta renda. Com relação aos adolescentes, a proporção que não atinge a recomendação diária de 60 minutos de atividade física moderada a vigorosa é 80,3%, sendo os meninos mais ativos que as meninas.

Dados publicados pelo Ministério da Saúde sobre a prática de atividade física em adultos brasileiros mostram que a frequência de atividade física suficiente no tempo livre (pelo menos 150 minutos semanais de atividade física de intensidade leve ou moderada ou pelo menos 75 minutos semanais de atividade física de intensidade vigorosa) é de 33,8%, sendo maior entre os homens (41,2%) do que entre as mulheres (27,4%) (Brasil, 2014). Já entre os adolescentes, os resultados da PeNSE 2012 mostram que 20,2% dos escolares praticam 60 minutos de atividade física em pelo menos cinco dias por semana, sendo 27,9%, para os estudantes do sexo masculino, e 13,1%, para os do sexo feminino (IBGE, 2012).

Um grande número de estudos tem avaliado a eficácia de programas de exercícios físicos na prevenção primária e secundária da obesidade. Esses programas são compostos por diferentes estruturas metodológicas, onde o exercício físico isoladamente ou em combinação com a dieta é avaliado (Farias, et al., 2009; Vasques, et al., 2014). Outras abordagens comumente testadas incluem a participação dos pais, a realização de atividades extracurriculares ou a recuperação de espaços públicos para a prática de atividades físicas (Ziebarth, et al., 2012; Branscum & Sharma, 2012; Puder, et al., 2011).

Os primeiros estudos para prevenção primária da obesidade em escolares seguiram as bases dos estudos para prevenção de doença cardiovascular. Dois grandes estudos intitulados *Pathways* (Lohman, et al., 2003) e *Planet Health* (Gortmaker, et al., 1999) foram conduzidos em escolares norte-americanos no final da década de 90 e suas intervenções incluíram tanto a pratica de atividades físicas quanto orientações sobre o consumo alimentar. Mesmo tendo sido conduzidos em populações com alta prevalência de obesidade, nenhum dos trabalhos demonstrou redução global na prevalência de sobrepeso e obesidade, resultado semelhante ao encontrado por outros estudos como o *New Moves* (Neumark-Sztainer, et al., 2003).

Entretanto, quanto extratificados por sexo, o estudo *Planet Health* demonstrou uma redução pequena, porém, estatisticamente significativa na prevalência de obesidade entre as meninas, após um período de 2 anos de seguimento, (de 23,6 para 20,3%). Mais recentemente, em uma revisão sistemática sobre programas de prevenção de ganho de peso em adolescentes, Kropski et al., (2008) observaram que apesar de não terem sido encontradas reduções do peso corporal em 6 dos 14 artigos incluídos no estudo, mudanças no comportamento alimentar e na prática de atividade física foram observadas.

Alguns pesquisadores argumentam que, mesmo que sem impacto sobre ganho de peso, mudanças comportamentais por si só justificam-se considerando que o problema da obesidade é muito complexo e não se consegue superar os muitos fatores que concorrem para a crescente epidemia de obesidade observada nos últimos anos (Kelly et al., 2008). Além disso, a maioria das intervenções consegue abordar somente uma fração muito pequena dos fatores que geram a obesidade (Silveira et al., 2011).

Por outro lado, outros estudos têm demonstrado que a pratica de atividade física pode ocasionar resultados positivos no controle ponderal. O estudo *Kiss* avaliou o efeito de um programa de atividade física de base escolar na aptidão cardiorrespiratória e adiposidade em 502 crianças de 6 e 11 anos de idade em duas províncias na Suíça. Os autores propuseram um modelo de intervenção com duração de 9 meses, combinando aumento da frequência semanal de aulas de Educação Física de 3 para 5 vezes por semana, com duração de 45 minutos por sessão; 3 a 5 sessões por dia de 2 a 5 minutos de exercícios de coordenação e equilíbrio nos intervalos das aulas e, ainda, 10 minutos diários de exercícios em casa. Os resultados mostraram aumento dos níveis de atividade física e da aptidão cardiorrespiratória e redução dos ganhos de adiposidade quando comparados ao grupo controle (Kriemler, et al., 2010).

Corroborando esses achados, o projeto *FITKids* investigou o efeito de 9 meses de intervenção em atividade física na aptidão cardiorrespiratória e adiposidade em crianças pré-púberes. Foram randomizadas 220 crianças de 8 a 9 anos para o grupo intervenção e grupo controle. A intervenção consistia na realização de 70 minutos de atividade física moderada a intensa, 5 vezes por semana. Tanto as crianças eutróficas quanto aquelas com sobrepeso pertencentes ao grupo intervenção aumentaram a aptidão cardiorrespiratória e reduziram o percentual de gordura (Khan, et al., 2014).

As revisões sobre o efeito da atividade física sobre o controle ponderal publicadas nos últimos anos têm encontrado resultados bastante controversos. Em meta-análise conduzida por Harris et al., (2009), intervenções de atividade física com base escolar parecem não ter impacto no IMC de crianças e adolescentes. Alinhada a esse estudo, uma revisão sistemática muito bem conduzida por Dobbins et al., (2013) também não foi capaz de observar impacto no IMC de crianças e adolescentes de 6 a 18 anos em consequência da realização de programas de atividade física desenvolvidos na escola. Entretanto, em revisão sistemática publicada por Vasconcellos et al., (2014), foram analisados 24 ensaios que investigaram o efeito da atividade física, isolada ou combinada com outro tipo de intervenção, na capacidade aeróbia, composição corporal, variáveis hemodinâmicas e marcadores bioquímicos em adolescentes com excesso de peso. Os resultados sugerem que intervenções com atividades físicas podem ser capazes de melhorar a aptidão física, composição corporal e diversos outros fatores de risco para doença cardiovascular em adolescentes obesos.

Na tentativa de entender os achados conflitantes encontrados na literatura, Metcalf et al., (2012), em revisão sistemática de estudos de prevenção baseados em atividade física, argumenta que os estudos conseguiram efeitos muito pequenos no aumento da atividade física, da ordem de aproximadamente 4 minutos de caminhada ou corrida por dia, o que poderia explicar, pelo menos em parte, a ausência de efeito dessas intervenções na redução ou manutenção do IMC em crianças. Assim, a importância do exercício físico na prevenção bem como para o processo de redução do peso corporal, mais especificamente da gordura corporal, em adolescentes com excesso de peso, ainda não está bem definido.

Esta inconsistência encontrada nos resultados pode ser parcialmente explicada pelo curto período de tempo das intervenções, ampla variedade metodológica na avaliação da composição corporal e, ainda, pelas diferentes estruturas dos protocolos de exercício empregadas nas intervenções (tipo de exercício empregado, duração e intensidade das sessões), além do fracasso das intervenções na tentativa de aumentar o tempo de prática de exercícios físicos.

- 1. **Efeito do exercício físico no gasto energético e na quantidade de atividade física diários**

A complexidade da regulação do peso corporal representa um dos maiores desafios para o entendimento da etiologia, tratamento e prevenção da obesidade. Apesar de intensamente estudada nos últimos anos, ainda apresenta muitas controvérsias, entre elas a sua relação com o exercício físico e gasto energético total (Roth, et al., 2004).

O gasto energético total (GET) pode ser dividido em três componentes: o gasto energético basal (GEB), necessário para a realização das funções vitais do organismo; o efeito térmico dos alimentos (ETA), relacionado com a digestão, absorção e o metabolismo dos alimentos; e o gasto energético da atividade física (GEAF) que engloba as atividades físicas do cotidiano e o exercício físico. O GEB corresponde a aproximadamente 60% a 75% do gasto energético diário, o ETA entre 5% e 15% e o GEAF de 15% a 30%, sendo este último o componente mais modificável (McArdle et al., 2008). Assim, o exercício físico vem sendo considerado há alguns anos como um potencial contribuidor para o aumento do gasto energético total, entretanto, resultados opostos também têm sido encontrados.

Van der Heijden et al., (2010) testaram a hipótese do gasto energético de 24 horas e a oxidação de gordura aumentarem em adolescentes magros e obesos após treinamento moderado. Foram selecionados 43 adolescentes magros e obesos para participarem de um programa de exercícios aeróbios, quatro vezes por semana e frequência cardíaca acima de 70% do consumo máximo de oxigênio (VO_2 máx_). Após 12 semanas de treinamento, o programa de exercícios proposto não foi capaz de aumentar o gasto energético total. A oxidação de gordura no período de 24 horas aumentou nos adolescentes magros, entretanto não sofreu alteração nos obesos. Vale ressaltar neste estudo a classificação da intensidade como moderada para uma frequência cardíaca acima de 70% do VO_2 máx_. De acordo com a literatura, a intensidade moderada é classificada para o intervalo de 46% a 63% do VO_2 máx_ e a intensidade vigorosa para o intervalo 64% a 90% do VO_2 máx_ (Physical Activity Guidelines Advisory Committee, 2008).

Esses resultados empíricos que contrariam o esperado papel do aumento da atividade física vêm sendo debatidos pela comunidade científica como um efeito compensatório que o exercício físico pode promover nas atividades físicas subsequentes. Os primeiros pesquisadores a abordarem esse fenômeno foram Epstein & Wing (1980), que em estudo de meta-análise sobre o efeito de exercícios aeróbios na perda de peso e gordura corporal observaram que os sujeitos que eram submetidos à sessões de exercícios apresentavam perda de peso abaixo do esperado. Os autores apresentaram duas possibilidades para esse acontecimento: a primeira era que o exercício físico estimulava o apetite, com aumento do consumo calórico; a segunda hipótese seria de que indivíduos que se exercitavam apresentavam o nível de atividade física modificado no restante do dia, ou seja, movimentavam-se menos devido a um suposto “cansaço” induzido pelo exercício, contribuindo, assim, para uma manutenção ou mesmo uma redução do gasto energético total. A partir daí, na década de 90, começam a ser publicados os primeiros estudos que testaram o efeito compensatório provocado pelo exercício físico no gasto energético diário.

Meijer et al., (1991) recrutaram 32 adultos, ambos os sexos, para um programa de treinamento visando a participação em uma meia maratona. O treinamento foi desenvolvido ao longo de 20 semanas e com uma frequência de 4 sessões por semana. A duração média de cada sessão era de 60 minutos e a intensidade variava de 70% a 100% da frequência cardíaca máxima, de acordo com o tipo de treinamento empregado. Após 8 semanas de treinamento, a distância semanal percorrida foi 15 a 25 km e, após 20 semanas, 25 a 40 km. Os resultados do estudo apontam que o treinamento físico aumentou a quantidade de atividade física desenvolvida, medida com acelerômetros, na vigésima semana em homens e mulheres (62% e 63%, respectivamente), quando comparada aos valores obtidos na linha de base e, ainda, o gasto energético total diário foi 3 a 4 vezes maior que o gasto despendido na sessão de exercício, somente nos homens. Portanto, um alto volume de treinamento associado a uma intensidade vigorosa é capaz de aumentar o a quantidade diária de atividade física em adultos, não sustentando a hipótese de efeito compensatório.

Posteriormente, Goran & Poehlman (1992), avaliaram o efeito do exercício aeróbio no gasto energético total em idosos saudáveis. Os exercícios foram executados em bicicleta ergométrica, 3 vezes por semana, durante 8 semanas. Após o treinamento, o gasto energético total, avaliado pelo método de água duplamente marcada, não sofreu alteração, quando comparado ao período pré-treinamento. Mesmo com o aumento no gasto energético basal e com o dispêndio energético promovido pelas sessões de exercício, os indivíduos se tornaram menos ativos ao longo do dia, apresentando uma redução no gasto energético promovido pelas atividades físicas espontâneas. Os autores argumentam que os resultados encontrados podem estar relacionados à intensidade vigorosa que os indivíduos foram expostos nas últimas semanas do treinamento, entretanto, esses resultados são similares aos encontrados em outros estudos que avaliaram o efeito de sessões de exercício físico moderado em indivíduos na mesma faixa etária (Meijer, et al., 1999; Meijer, et al., 2000).

Blaak et al., (1991) foram os primeiros a investigarem a hipótese do efeito compensatório provocado pelo exercício físico em crianças. Neste estudo, foram selecionados 10 meninos obesos de 10 a 11 anos de idade para participarem de um programa de treinamento aeróbio por 4 semanas. Os sujeitos realizavam exercício em cicloergômetro, 5 vezes por semana, durante 60 minutos em intensidade moderada. Os resultados do estudo indicam que adicionar 1 hora de exercício físico por dia proporciona um aumento significativo no gasto energético diário em meninos obesos e que alterações na prática de atividades físicas espontâneas não foram observadas.

Na tentativa de explicar o fenômeno do efeito compensatório, Rowland (1998) descreve pela primeira vez a hipótese do “*Activitystat*”, definindo como um mecanismo homeostático onde um centro de controle biológico seria o responsável pelo controle da atividade física de acordo com um *set point* de gasto energético. É um mecanismo que permite a continuidade da estabilidade em um sistema dinâmico através de um processo de *feedback* negativo. Sempre que ocorre um desequilíbrio, sistemas regulatórios tornam-se ativos para restaurar a linha de base (Guyton & Hall, 2006). De acordo com essa teoria, o aumento ou a diminuição dos níveis de atividade física em um determinado momento seria compensado com a alteração destes níveis em outro momento, em defesa de um *set point* individual. O tipo, a duração e intensidade dos exercícios físicos são componentes importantes a serem considerados no processo de investigação dessa hipótese.

Dale et al., (2000) foram os primeiros a publicarem dados com referência específica ao *Activitystat*. De acordo com o estudo, a restrição de atividade física imposta no período escolar não foi compensada com o aumento de atividade física no período fora da escola. Quatro anos mais tarde, Metcalf et al., (2004), utilizando acelerômetros, compararam a quantidade de atividade física realizada na semana entre aqueles que iam a pé para a escola e aqueles que eram levados de carro. Apesar da quantidade de atividade física semanal durante o período de deslocamento ser maior entre aqueles que iam a pé, quando avaliado todo o período do dia essa diferença não era sustentada. A partir deste trabalho, outros foram publicados avaliando esta hipótese, tanto em adultos quanto em crianças.

Por outro lado, Wilkin et al., (2006) demonstraram em crianças de 9 anos de idade que a quantidade total de atividade física desenvolvida era similar entre aquelas crianças que frequentavam escolas com um alto volume de aulas de Educação Física comparado aquelas que possuíam poucas aulas, sugerindo que aquelas crianças que possuíam poucas aulas realizavam mais atividades no período subsequente, de tal forma que a diferença que existia inicialmente era compensada no final. Corroborando esses achados, Fremeaux et al., (2011), em estudo semelhante, avaliaram se um maior volume de atividades físicas no período escolar poderia ser compensado com menor volume de atividades físicas no período fora da escola. Foram selecionadas para participar do estudo 206 crianças de três escolas com diferentes volumes de aulas de Educação Física. Os dados de atividade física foram obtidos com acelerômetros, onde os participantes foram orientados a utilizar por 4 semanas. Os resultados mostram que a prática aumentada de atividade física escolar induzia uma redução nas atividades físicas espontâneas fora da escola, não apresentando, portanto, diferença significativa na quantidade de atividade física semanal entre as escolas. Contudo, outros trabalhos que avaliaram os efeitos de diferentes volumes de atividades físicas sobre as atividades subsequentes encontraram resultados diferentes.

A grande variedade metodológica empregada pode ser um dos motivos para as diferenças encontradas nos resultados dos estudos. Um programa de exercícios físicos deve ser estruturado levando em consideração alguns fatores, como: o tipo de exercício físico (aeróbio, resistido ou flexibilidade), a duração, a frequência semanal e a intensidade da atividade (leve, moderada ou vigorosa), podendo cada uma destas variáveis ou as combinações possíveis entre elas, influenciar de diferentes formas os diversos desfechos avaliados.

Com relação à intensidade, acreditava-se que exercícios de intensidade leve seriam os mais apropriados a serem prescritos para indivíduos obesos, devido, principalmente, a proporção de lipídeos oxidados durante a atividade física ser maior nessa zona de intensidade (Achten, et al., 2002). Entretanto, atualmente acredita-se que o gasto energético total (GET) e o balanço energético negativo devem ser o foco quando o objetivo principal é a redução de peso. A zona ideal de intensidade do exercício físico para a otimização do GET e consequente redução da gordura coporal continua sendo discutida na literatura.

Em estudo transversal, Tremblay et al., (1990) avaliaram o efeito de diferentes intensidades de atividades físicas na gordura corporal. Foram analisados dados de atividade física e gordura corporal de 2623 indivíduos adultos, de ambos os sexos, que participaram do inquérito de aptidão física canadense em 1981 (Stephens, et al., 1986). A atividade física foi avaliada através de questionários e a gordura corporal pelo método de dobra cutânea. Os autores concluiram que o grupo que reportou praticar atividades físicas intensas com regularidade por um período superior a 6 meses apresentou menor percentual de gordura corporal e, ainda, menor relação cintura-quadril. Sugerem, portanto, que o aumento da intensidade do exercício pode favorecer um balanço energético negativo.O mesmo pesquisador, quatro anos mais tarde, realizou outro estudo para avaliar o impacto da intensidade do exercício físico no peso e na gordura corporal através de um programa de exercício em cicloergômetro por 15 a 20 semanas. Foram selecionados 27 adultos saudáveis, de ambos os sexos, para compor os grupos de intensidade moderada e intensa. Apesar do peso corporal não ter apresentado alteração, a gordura subcutânea foi significativamente menor no grupo que desenvolveu o protocolo de alta intensidade (Tremblay, et al., 1994). Nessa mesma linha, Trapp et al., (2008) compararam os efeitos do exercício intermitente de alta intensidade com os do exercício moderado contínuo, realizados por 15 semanas, em 45 mulheres saudáveis. Ambos os grupos apresentaram aumento na aptidão cardiovascular, no entanto, somente o grupo de alta intensidade apresentou redução significativa do peso corporal total, peso de gordura e níveis plasmáticos de insulina.

Por outro lado, diversos outros estudos não apresentam diferenças em seus resultados quando comparam o efeito de diferentes intensidades do exercício no peso ou gordura corporal. Por exemplo, Jakicic et al., (2003) selecionaram 201 mulheres com sobrepeso e sedentárias e randomizaram para quatro diferentes programas de exercícios, combinando intensidade e duração: 1) intensidade vigorosa/ longa duração, 2) intensidade moderada/ longa duração, 3) intensidade moderada/ moderada duração e 4) intensidade vigorosa/ moderada duração. Os participantes foram orientados a realizar o programa cinco vezes por semana e todos receberam orientação nutricional. Após 12 meses de treinamento, foram observadas redução no peso corporal e melhora na aptidão cardiorrespiratória em todos os grupos estudados, não tendo sido encontradas diferença entre os grupos.

Em outro estudo, Duncan et al., (2005) compararam os efeitos da caminhada em diferentes intensidades e frequência semanal na aptidão cardiorrespiratória e no perfil lipídico em 492 adultos saudáveis. O protocolo experimental contemplava quatro diferentes condições: intensidade moderada/ baixa frequência, intensidade moderada/ alta frequência, intensidade alta/ baixa frequência e intensidade alta/ alta frequência. A zona de treinamento foi determinada em 45% a 55% e 65% a 75% da frequência cardíaca de reserva para intensidades moderada e intensa, respectivamente. Os participantes do grupo de baixa frequência semanal foram orientados a caminhar de 3 a 4 vezes por semana enquanto o grupo de alta frequência de 5 a 7 dias por semana. Após 24 meses, nenhum dos grupos apresentou alteração significativa no peso corporal. Tjonna et al., (2008) examinaram os efeitos do exercício físico moderado *versus* o intenso em variáveis associadas à função cardiovascular em 32 pacientes com síndrome metabólica e concluíram que os dois programas de exercício foram igualmente eficazes em reduzir a pressão arterial, o peso e a gordura corporal.

Wang et al., (2011) selecionaram 36 mulheres obesas pós-menopausa e randomizaram em dois grupos. O primeiro grupo foi orientado a realizar caminhada na esteira numa intensidade moderada (45% - 50% do consumo máximo de oxigênio) enquanto o segundo grupo numa intensidade vigorosa (70% – 75% do consumo máximo de oxigênio), ambos com frequência de 3 vezes por semana. O estudo teve duração de cinco meses e os dois grupos também receberam orientação nutricional. Durante o último mês do estudo, o gasto energético entre as mulheres do grupo que realizou atividade moderada foi maior nos dias em que realizavam o exercício (577,7 ± 219,7 kcal.d^-1^) comparado aos dias em que não realizavam (450,7 ± 140,5 kcal.d^-1^), no entanto, a diferença encontrada era muito menor do que a gasta com as sessões (127,0 ± 188,1 kcal.d^-1^). Nas mulheres que realizaram o exercício vigoroso, o gasto energético nos dias com exercício (450,6 ± 153,6 kcal.d^-1^) era menor comparado aos dias sem exercício (519,2 ± 127,4 kcal.d^-1^). Os autores concluem que existe um efeito compensatório promovido pelo exercício físico que ocorre principalmente quando atividades de intensidade vigorosa.

Kriemler et al., (1999) avaliaram o impacto de uma sessão de exercício físico no gasto energético e na quantidade de atividade física praticada por 14 adolescentes obesos. O estudo estabelecia a participação em 3 sessões: na primeira, os adolescentes deveriam realizar 4 *sets* de 10 minutos com intervalos de 5 minutos numa frequência cardíaca de 150 a 160 bpm (intensidade vigorosa); na segunda, foram orientados a executar 2 *sets* de 15 minutos com 5 minutos de intervalo numa frequência cardíaca de 130 a 140 bpm (intensidade moderada) e a terceira era a sessão controle onde os adolescentes não realizavam a sessão de exercício. As sessões foram realizadas em bicicleta ergométrica, no período da manhã e, para cada sessão experimental, o gasto energético e a quantidade de atividade física foram avaliados no dia anterior (d1), no dia da sessão (d2) e no dia seguinte (d3). Os autores concluem que após cada sessão de exercício, o gasto energético e a quantidade de atividade física apresentam-se reduzidos no período da tarde e que a média do gasto energético de d2 e d3 comparada ao gasto energético de d1 é dose dependente. A intensidade moderada promove aumento, entretanto, a intensidade vigorosa leva a redução das atividades físicas subsequentes.

Em trabalho recente comparando o efeito de diferentes intensidades do treinamento físico sobre o gasto energético diário em indivíduos eutróficos e obesos, Thivel et al., (2013) apresentou resultados de três estudos que observaram que, embora o exercício físico vigoroso possa contribuir para uma melhora na composição corporal, na aptidão física e no perfil metabólico (Boutcher, 2011), os adolescentes obesos parecem apresentar uma resposta compensatória a uma sessão de exercício de alta intensidade (acima de 70% do VO_2 max_) diminuindo o gasto energético das atividades físicas subsequentes, fato esse não observado nos adolescentes com peso normal.

Entretanto, os estudos que avaliam os efeitos de diferentes intensidades de exercício físico sobre o gasto energético diário bem como seus efeitos compensatórios nas atividades físicas espontâneas ainda são controversos, sendo necessários novos trabalhos nessa área.

1. **Justificativa**

O exercício físico tem sido considerado como uma estratégia importante em diversos programas de redução de obesidade, tanto em adultos quanto em crianças e adolescentes. Em alguns trabalhos, entretanto, a prática de exercícios físicos não tem contribuído de forma significativa para a redução do peso ou gordura corporal (Swift, et al., 2014). Esta inconsistência encontrada nos resultados pode ser parcialmente explicada pelo curto período de tempo das intervenções, ampla variedade metodológica empregada na avaliação da composição corporal e na constituição das intervenções (tipo de exercício empregado, frequência semanal, duração e intensidade das sessões de exercícios), onde cada um desses componentes pode interferir de forma diferente cada desfecho estudado.

Posicionamentos recentes no Brasil e no exterior têm recomendado a prática diária de exercícios físicos, com duração mínima de 60 minutos de intensidade moderada a vigorosa, tanto para a manutenção da saúde quanto para a prevenção da obesidade e outras doenças (Landry & Driscoll, 2012). Entretanto, especificamente para a redução de peso, a intensidade do exercício ainda é um ponto de discussão na literatura.

Enquanto alguns estudos observam maior perda de peso para aqueles que realizam exercício físico vigoroso (Tremblay, et al., 1994; Trapp, et al., 2008), outros não observam qualquer diferença quando o comparam com o exercício físico moderado (Jakicic, et al., 2003; Tjonna, et al., 2008). Assim, as diferentes zonas de intensidade parecem influenciar de forma diferenciada o gasto energético e a quantidade de atividade física diários.

Alguns pesquisadores recomendam fortemente a prática de exercícios físicos de alta intensidade para a perda de peso, enquanto outros acreditam que exercícios físicos moderados e a redução de atividades sedentárias devem ser incentivados como melhor alternativa para o aumento do gasto energético diário e para redução de peso corporal (Westerterp & Plasqui, 2004), principalmente, pelo fato de indivíduos obesos sentirem mais conforto e confiança com a prática de exercícios moderados (Piana, et al., 2013). Entretanto, poucos ainda são os estudos que avaliaram o efeito de diferentes intensidades do exercício físico no gasto energético diário em crianças com excesso de peso.

Portanto, a intensidade do exercício físico que deve ser recomendada para crianças e adolescentes para perda de peso ainda precisa ser esclarecida e o efeito compensatório provocado pelo exercício físico nas atividades físicas subsequentes ainda é uma hipótese a ser testada.

1. **Objetivos**
   1. **Objetivo Geral**

Avaliar o efeito de duas intensidades de exercício físico no gasto energético das atividades físicas espontâneas subsequentes em adolescentes com excesso de peso.

- 1. **Objetivos Específicos**

Avaliar e quantificar a existência de efeito compensatório do exercício físico nas atividades físicas subsequentes.

Comparar a quantidade de atividade física diária em adolescentes submetidos a diferentes protocolos de intensidade de treinamento.

1. **Métodos**
   1. **Desenho do estudo**

Caracteriza-se como um estudo experimental do tipo *crossover*, onde todos os sujeitos selecionados serão submetidos a três condições experimentais.

No início do ano letivo de 2014, todos os alunos matriculados nos 6 e 7 anos da Escola Municipal Honorina de Carvalho (Niterói- RJ) foram convidados a participar do projeto PAPPAS, desenvolvido pelo mesmo grupo de pesquisa. A massa corporal e a estatura foram aferidas e, com esses dados, foi  calculado o IMC. Foram considerados com excesso de peso os adolescentes que apresentaram valores de IMC para a idade superiores ao escore z + 1 (de Onis, et al., 2007). Uma amostra desses meninos com excesso de peso, selecionada aleatoriamente, será convidada a participar do estudo. O convite será feito individualmente e, antes do primeiro tempo de aula, todos serão reunidos em uma sala para que o projeto possa ser explicado. A massa corporal e estatura serão novamente aferidas e a classificação do estado nutricional confirmada. Como forma de reduzir a exposição, as sessões experimentais serão realizadas após o último tempo de aula (fora do turno escolar). Como forma de minimizar o risco de estigmatização, adolescentes eutróficos serão convidados a participar das sessões de exercício, com o quantitativo correspondente a 20% do tamanho amostral.

Para isso, os adolescentes serão avaliados em cinco visitas realizadas na escola em que estudam. Durante a primeira visita, serão coletadas as medidas antropométricas e de composição corporal bem como serão identificados possíveis fatores de exclusão para participação no estudo. Durante a segunda visita, os adolescentes serão submetidos a um teste de campo máximo para avaliação da capacidade aeróbia (Leger, et al., 1988). Ao final do teste, a frequência cardíaca máxima será determinada e, a partir daí, a zona da frequência cardíaca para cada sessão experimental será calculada individualmente. A terceira e quarta visitas serão as sessões experimentais com diferentes intensidades de treinamento e a quinta sessão será controle (sem exercício), intercaladas por um período de uma semana (**figura 3**).


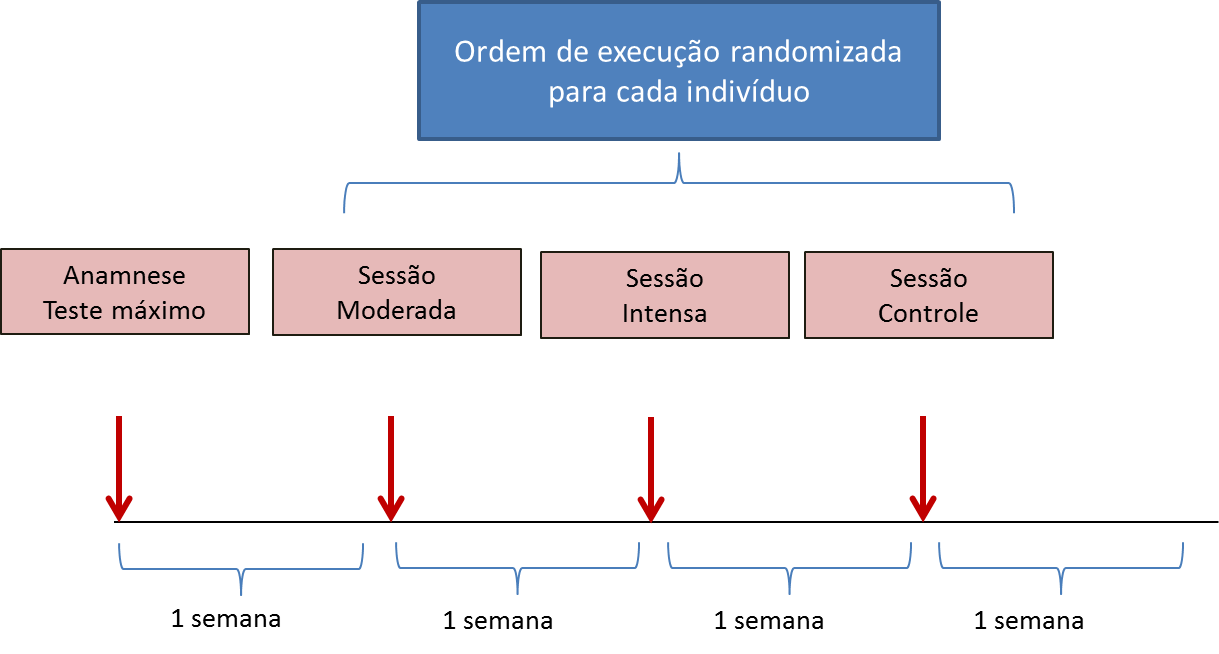


**Figura 3.** Esquema representativo das sessões experimentais

As duas sessões experimentais serão estruturadas em três fases: aquecimento, parte principal e volta a calma. A prescrição do treinamento será diferente entre os grupos experimentais apenas na parte principal, sendo as fases de aquecimento e a volta a calma idênticas nos dois grupos. Durante a fase de aquecimento, os sujeitos serão orientados a realizar uma caminhada, em intensidade leve, progredindo gradativamente a velocidade até alcançarem, ao final de 5 minutos, valores próximos a 64% da frequência cardíaca máxima (Fc_máx_). Na fase de volta a calma, com duração de 5 minutos, os adolescentes serão orientados a reduzirem gradativamente a velocidade da caminhada com o objetivo de atingirem valores de frequência cardíaca próximos aos valores encontrados durante o repouso. (**figura 4**).


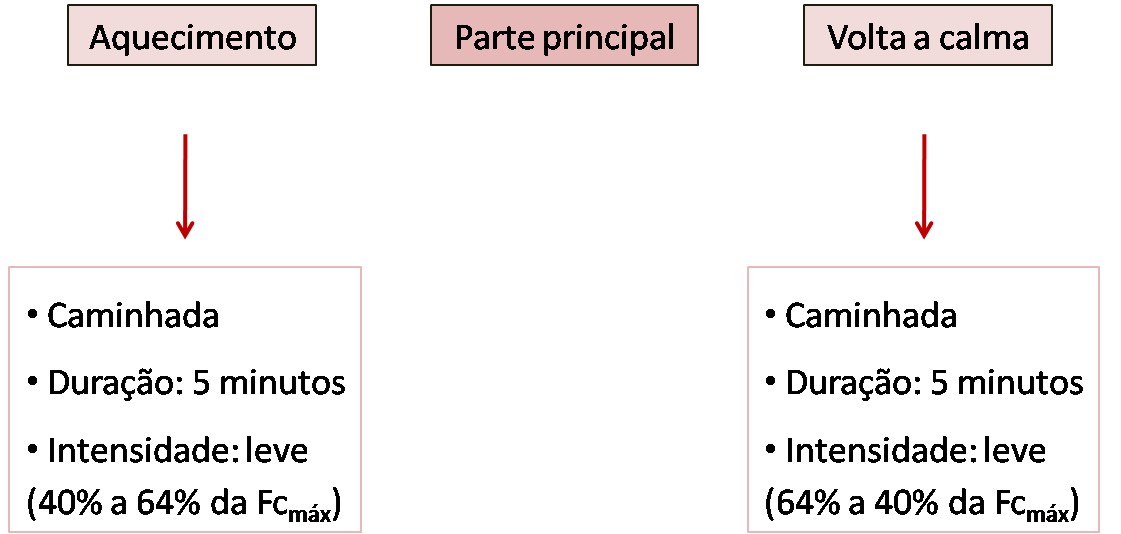


**Figura 4.** Descrição das características das fases **aquecimento** e **volta a calma** das duas sessões experimentais.

A parte principal da sessão de treinamento moderado (TM) será constituída por 4 *sets* (períodos) de 10 minutos de corrida e/ ou caminhada, em intensidade moderada (64% a 76% da frequência cardíaca máxima), com um intervalo entre os *sets* de 5 minutos de caminhada leve (abaixo de 64% da frequência cardíaca máxima) para a recuperação (**figura 5**).


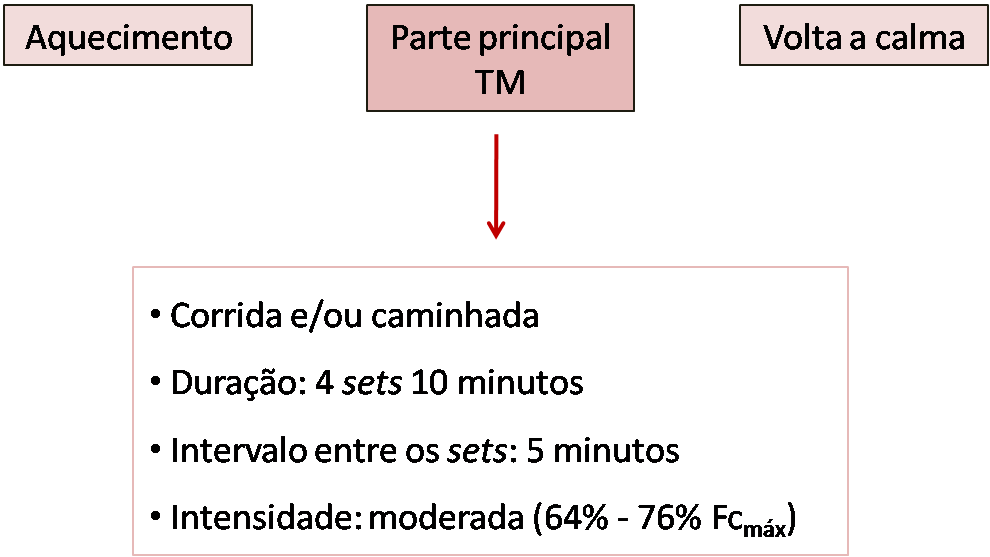


**Figura 5.** Descrição das características da fase **parte principal** da sessão moderada.

A parte principal da sessão de treinamento intenso (TI) será composta por 4 *sets* (períodos) de 10 minutos de corrida e/ ou caminhada, numa intensidade vigorosa (77% a 95% da frequência cardíaca máxima), com um intervalo entre os *sets* de 5 minutos de caminhada leve (abaixo de 64% da frequência cardíaca máxima) para a recuperação (**figura 6**).


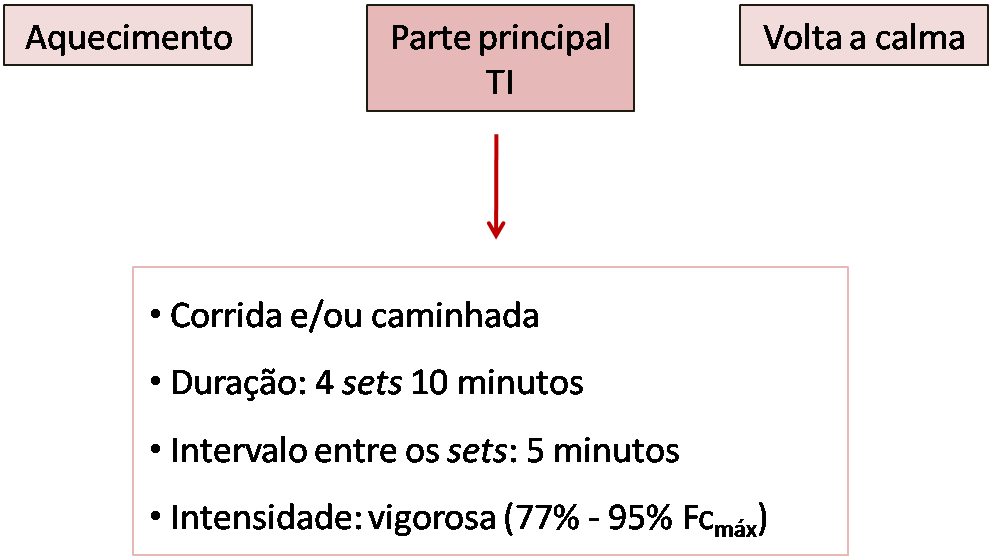


**Figura 6.** Descrição das características da fase **parte principal** da sessão vigorosa.

Durante as duas sessões experimentais, os adolescentes serão supervisionados por profissionais de educação física treinados para a realização do protocolo de intervenção. A zona alvo de frequência cardíaca será controlada pelos próprios adolescentes e também pelos profissionais que supervisionam a sessão de treinamento, através da utilização de frequencímetros. Será dado intervalo de uma semana entre as sessões de treinamento como período de *washout* (**figura 3**).

Durante as sessões experimentais e por um período de 6 dias, o gasto energético e a quantidade de atividade física serão avaliados através do uso de acelerômetros. Consiste em um pequeno aparelho que será posicionado na região do quadril e deverá ser retirado somente durante o banho ou durante a prática de atividades aquáticas. O uso deste aparelho é considerado seguro, entretanto a sua utilização por um período prolongado pode provocar desconforto. Todos os adolescentes serão orientados a não realizar exercícios físicos nos três dias que antecedem cada sessão e, não ingerir bebida alcoólica, café ou qualquer outro estimulante nas 24 horas anteriores a cada sessão.

- 1. **População do estudo**

Todos os meninos de 11 a 14 anos com excesso de peso matriculados na escola pública municipal selecionada serão convidados para participarem do estudo. Para aqueles que aceitarem, será aplicado o questionário PAR-Q (*Physical Activity Readiness Questionnaire*). Este questionário é composto por sete perguntas, entre as quais cinco estão relacionadas a sintomas cardiovasculares. De acordo com os idealizadores do instrumento (Shephard, et al., 1981), o indivíduo que responder negativamente a todas as questões está apto a iniciar a prática do exercício físico sem necessitar de avaliação médica. Aqueles que referirem diabetes, doença cardiovascular ou alguma lesão osteomioarticular que impeça a realização das atividades, serão excluídos do estudo (American College of Sports Medicine, 2009).

O cálculo do tamanho da amostra baseou-se em uma diferença do gasto energético diário de 110 kcal (Wang, et al., 2006) entre os grupos de intervenção, com coeficiente de variação igual a 1, ou seja, desvio padrão igual a 110 kcal (van Belle, 2008). Utilizando um α de 0,05 e β de 0,10 e estimando uma taxa de recusas de 20%, o tamanho de amostra necessário para realização do estudo é de 27 adolescentes, que serão selecionados aleatoriamente (Julious, 2009).

- 1. **Monitoramento e Avaliação**
     1. **Avaliação antropométrica**

A massa corporal será aferida uma única vez em balança eletrônica (Tanita) com resolução de 50g, com roupas leves e sem sapatos. A estatura será medida em estadiômetro portátil da marca Alturexata, com resolução de 0,1 cm que foi posicionado em uma parede sem rodapé. Os participantes serão orientados a permanecer descalços, com os braços ao longo do corpo, pés unidos e com a cabeça, nádegas e calcanhares encostados na parede, mantendo a cabeça no plano de *Frankfurt* (Madsen, et al., 2008). A régua do estadiômetro será então deslocada até a cabeça do adolescente e realizada a leitura após uma expiração normal. Serão feitas duas aferições da estatura e será considerada a média dos valores para as análises. Com esses dados, será  calculado o IMC através da divisão da massa corporal (kg) pela estatura elevada ao quadrado (m^2^). A classificação do estado nutricional será realizada com base nos critérios da OMS. Serão considerados com excesso de peso os adolescentes que apresentarem valores de IMC para a idade superiores ao escore z + 1 (de Onis, et al., 2007).

- - 1. **Avaliação da capacidade aeróbia máxima**

A aptidão cardiorrespiratória será avaliada através de teste máximo de campo, específico para a faixa etária do estudo. O teste que será utilizado é o *ShuttleRun Test* (Teste de corrida de vai e vem de 20 metros), validado (Leger, et al., 1988) e amplamente empregado em estudos com adolescentes em todo o mundo (Jimenez-Pavon, et al., 2013; Veses, et al., 2014). O objetivo deste teste é estimar o consumo máximo de oxigênio (VO_2máx_), permitindo avaliar a capacidade aeróbia dos adolescentes. É um teste do tipo progressivo, máximo e indireto e consiste em realizar percursos de 20 metros, em regime de vai e vem, a uma velocidade imposta por sinais sonoros (provenientes de uma gravação do protocolo do teste). O teste inicia-se a uma velocidade de 8,5 km.h^-1^ e é constituído por patamares de um minuto, com o aumento da velocidade e, consequentemente, aumento do número de percursos em cada patamar. Os participantes colocam-se na linha de partida e iniciam o teste ao primeiro sinal sonoro. Deverão chegar ao local marcado, ultrapassando a linha, antes de soar o próximo sinal sonoro. As mudanças de direção devem ser feitas com parada e arranque para o lado contrário, evitando trajetórias curvilíneas. Em cada patamar (cada minuto), o intervalo de tempo entre os sinais sonoros vai diminuindo, o que significará um aumento da velocidade de execução dos participantes (0,5 km.h^-1^ por patamar). O teste será finalizado com a desistência do participante, ou quando este não conseguir atingir a linha demarcada duas vezes consecutivas. Deve ser controlado e registrado o número de percursos completos realizado por cada participante, excluindo o percurso no qual foi interrompido o teste. Ao final do teste, os participantes deverão realizar uma caminhada leve por pelo menos 3 minutos, facilitando a volta a calma. Um médico estará presente durante todo o período de execução do teste para eventuais intercorrências.

Cada participante utilizará um frequencímetro durante o teste e sua frequência cardíaca máxima será registrada. Assim, a intensidade de cada sessão de exercício (moderada e vigorosa) será calculada, individualmente, de acordo com o percentual da frequência máxima estabelecida para as intensidades moderada e vigorosa (Physical Activity Guidelines Advisory Committee, 2008).

- - 1. **Avaliação do gasto energético e da quantidade de atividade física**

O gasto energético promovido pelas atividades físicas e a quantidade de atividade física espontânea desenvolvida durante uma semana serão avaliados através de acelerômetros triaxiais da marca *Actigraph*, modelo wGT3X-BT. É um aparelho portátil, leve e não invasivo capaz de identificar as acelerações produzidas pelo corpo humano, proporcionando uma medição objetiva das atividades físicas desenvolvidas. Pode ser utilizado em diferentes regiões do corpo (coluna lombar, tornozelo, punho e coxa), entretanto, para maior acurácia, deve ser posicionado no quadril (Cleland, et al., 2013). O lado direito será o escolhido por ser o lado dominante da maioria dos adolescentes. O acelerômetro será colocado um dia antes de cada sessão experimental, no mesmo horário para todas as duas sessões e retirado uma semana após a realização do exercício.

Frequentemente utilizados em estudos epidemiológicos quando se pretende avaliar as recomendações para a prática de atividade física ou a quantidade de atividade física espontânea em crianças e adolescentes, além de fornecer uma estimativa do gasto energético proveniente das atividades realizadas. Como forma de proceder com as avaliações, foram desenvolvidos valores limítrofes com o objetivo de fracionar a intensidade em categorias: sedentária, leve, moderada e intensa.

A análise dos dados será realizada por *epochs* (período de amostragem), sendo contabilizado o número de impulsos por minuto (*counts/min*) e classificada a categoria de intensidade correspondente (sedentária, leve, moderada e vigorosa) em função dos valores limítrofes definidos. Os pontos limítrofes que serão utilizados para cada para cada faixa de intensidade foram propostos por Evenson (2008), onde abaixo de 100 impulsos por minuto a atividade física é classificada como sedentária, entre 100 e 2296 impulsos por minuto como atividade leve, entre 2296 e 4012 como atividade moderada e acima de 4012 atividade física vigorosa. Esses pontos mostraram maior precisão para classificar a intensidade da atividade física do que outros pontos estabelecidos em outros estudos (Trost, et al., 2011).

Esses períodos de tempo serão posteriormente acumulados de modo a fornecerem indicações sobre o tempo despendido pelo sujeito em diferentes níveis de intensidade para o dia de registro. O período de amostragem (*epoch*) neste estudo será de 5 segundos para a possibilidade de captação de pequenas variações de movimentos realizados pelos adolescentes em condições habituais (Trost, et al., 2005). Os dias com menos de 600 minutos de dados registrados e períodos superiores a 60 minutos de *counts* de zeros consecutivos, serão excluídos das análises (Choi, et al., 2011).

Os adolescentes serão orientados a não retirarem o aparelho durante todo esse tempo, com exceção para o período do banho e durante a prática de atividades aquáticas.

- 1. **Análise dos dados**

Serão realizadas estimativas de média e desvio-padrão das variáveis contínuas investigadas no estudo. A comparação das variações das médias de gasto energético e quantidade de atividade física diária entre os dois protocolos de exercício serão realizadas por meio de modelos lineares mistos (PROC MIXED), que levam em conta a correlação entre medidas repetidas no tempo.

Todas as análises estatísticas serão realizadas utilizando-se o programa SAS versão 9.3 (SAS Institute Inc, Cary, NC) e para todos os testes estatísticos, considerou- se significante valor de p < 0,05.

1. **Cronograma**

**
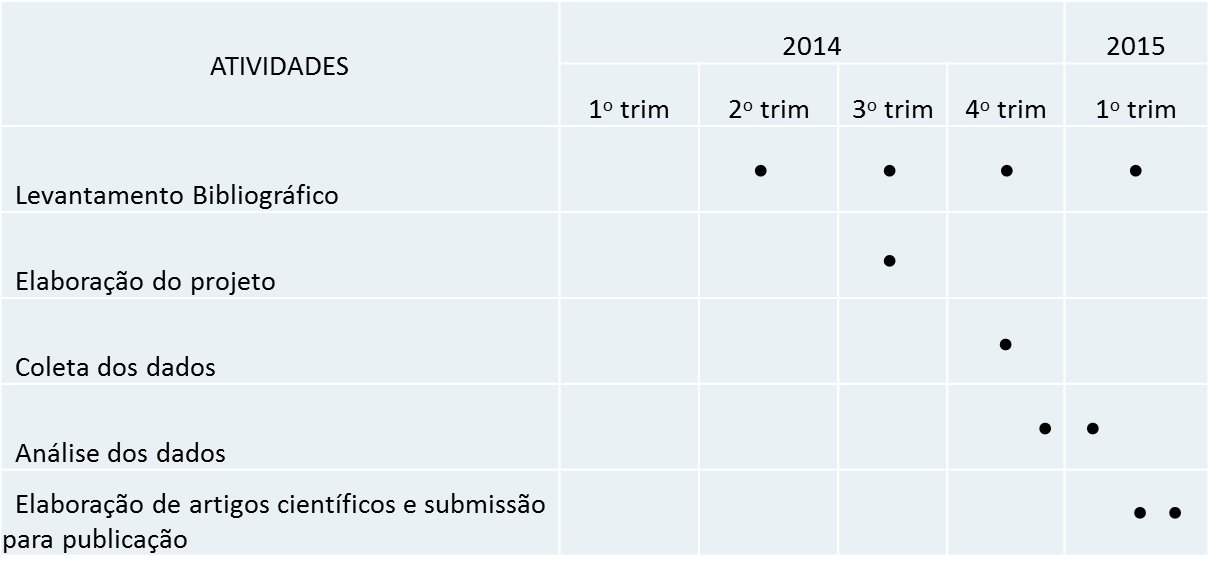
**

•

•

•

•

1. **Referências Bibliográficas**

Achten J, Gleeson M, Jeukendrup A. Determination of the exercise intensity that elicits maximal fat oxidation. Med Sci Sports Exerc 2002; 34: 92-97.

American College of Sports Medicine. ACSM’ Guidelines for exercise testing and prescription. 8 ed. Lippincott Willians & Wilkins, Philadelphia, 2009.
