# Supplementary material for: Effect of Exercise Intensity on Spontaneous Physical Activity Energy Expenditure in Overweight Boys: A Crossover Study
Source: PLoS One. 2016 Jan 15;11(1):e0147141. doi: 10.1371/journal.pone.0147141 (PMC4714875; doi:10.1371/journal.pone.0147141)
Supplement: S2 Protocol — (DOC) [file pone.0147141.s003.doc]

| 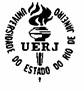 **No** | **UNIVERSIDADE DO ESTADO DO RIO DE JANEIRO**  **Instituto de Medicina Social**  Rua São Francisco Xavier, 524 / 7º andar / Blocos D e E - Maracanã  CEP: 20559.900 - Rio de Janeiro - RJ - BRASIL  TELS: 55-21-587-73 03/587-7540/587-7422/587-7572/284-8249  FAX: 55-21-264-1142  [http://www.ims.uerj.br](http://www.ims.uerj.br/)  ­­­­­ | 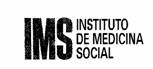 |
| --- | --- | --- |

Research project: Effects of different intensities of exercise on daily energy expenditure with physical activity in overweight boys

This is a master's project, approved on the qualify exam July 29, 2014.

1. **Background**
   1. **Prevalence of obesity, determinants e consequences**

Changes associated with modernization and industrialization undergone by society in recent years reflect directly in major changes in the pattern of physical activity and nutrition of the population. As a result of these changes, a rapid increase in the prevalence of overweight and obesity not only in adults but also in children and adolescents has been observed (de Onis, et al, 2010;.. Swinburn, et al, 2011; Wang & Lim , 2012).

It is estimated that 1.46 billion adults worldwide are overweight, and of these, 502 million are obese (Finucane et al., 2011). In addition, data from the World Health Organization (WHO) shows that approximately 20% of children and adolescents in Western countries already have overweight/obesity (Saha et al., 2011).
In Brazil national surveys trend shows marked decline in the prevalence of malnutrition and a significant increase of overweight and obesity (Batista Filho & Rissin 2003).

The Brazilian Household Budged Survey (POF) 2008-2009 has shown that being overweight affects 50.1% of men and 48.0% of women and that the prevalence of obesity reached 12.4% among men and 16.9% among women. Comparing the period 1974/1975 to 2008/2009, overweight has nearly tripled among men (from 18.5% to 50.1%) and among women was almost twice as high (28 7% to 48.0%), whereas the prevalence of obesity increased by more than four times for men (2.8% to 12.4%) and more than twice for women (8.0% to 16.9%) (IBGE , 2010).

The 2013 surveillance risk factors survey by telephone Interviews (VIGITEL) confirmed the upward trend in the prevalence of overweight, especially in males. Adults of 27 cities in Brazil had a frequency of overweight of 50.8%, higher among men (54.7%) than among women (47.4%) (Brazil, 2014).

For children and adolescents, a systematic review of prevalence studies of overweight conducted in different regions of Brazil points to values ​​ranging from 4% to 31% (Araújo et al., 2012) with prevalence higher than 37 % in the most recent studies (Kaufmann & Albernaz, 2013; Brown et al, 2013). These prevalences indicate the relevance to public health system since a child or adolescent overweight are more likely to have this condition in adulthood, and also have low self-esteem, negative self-image, eating disorders and poor quality of life (Sharma, 2006; Herman, et al, 2009; Juhola, et al, 2011; Reilly & Kelly, 2011), with increased risk of comorbidities as hypertension, dyslipidemia, hiperinsulidemia, type 2 diabetes mellitus, sleep apnea, asthma and orthopedic complications (Kiess, et al. 2003;. Gidding, et al, 2004; Laska, et al. 2012; Raj, 2012).

Nutritional status is under both the genetic influence, environment influence and the interaction between them, but it is clear, that the high prevalence of inappropriate eating habits, increasing caloric density of the food, large portions of food consumed and the increased consumption of sweetened beverages (Ludwig, et al., 2001; Sichieri , 2013) associated with physical inactivity and sedentary habits are the major contributors to the increasing occurrence of obesity and other chronic non-communicable diseases.

The complexity of the factors associated with obesity is shown in Figure 1, which identify some of the potential determinants and their possible interrelationships among children (Monasta, et al., 2010). The review indicates that breastfeeding may be a protective factor for future obesity while childhood obesity, rapid infant growth, gestational diabetes, maternal smoking, little or too much sleep, less than 30 minutes of daily physical activity and consumption of sweetened beverages can be considered as important risk factors. Parental obesity, intrauterine growth and birth weight inadequate, television time, food insecurity and low socioeconomic status may also be included among the risk factors although it is difficult to


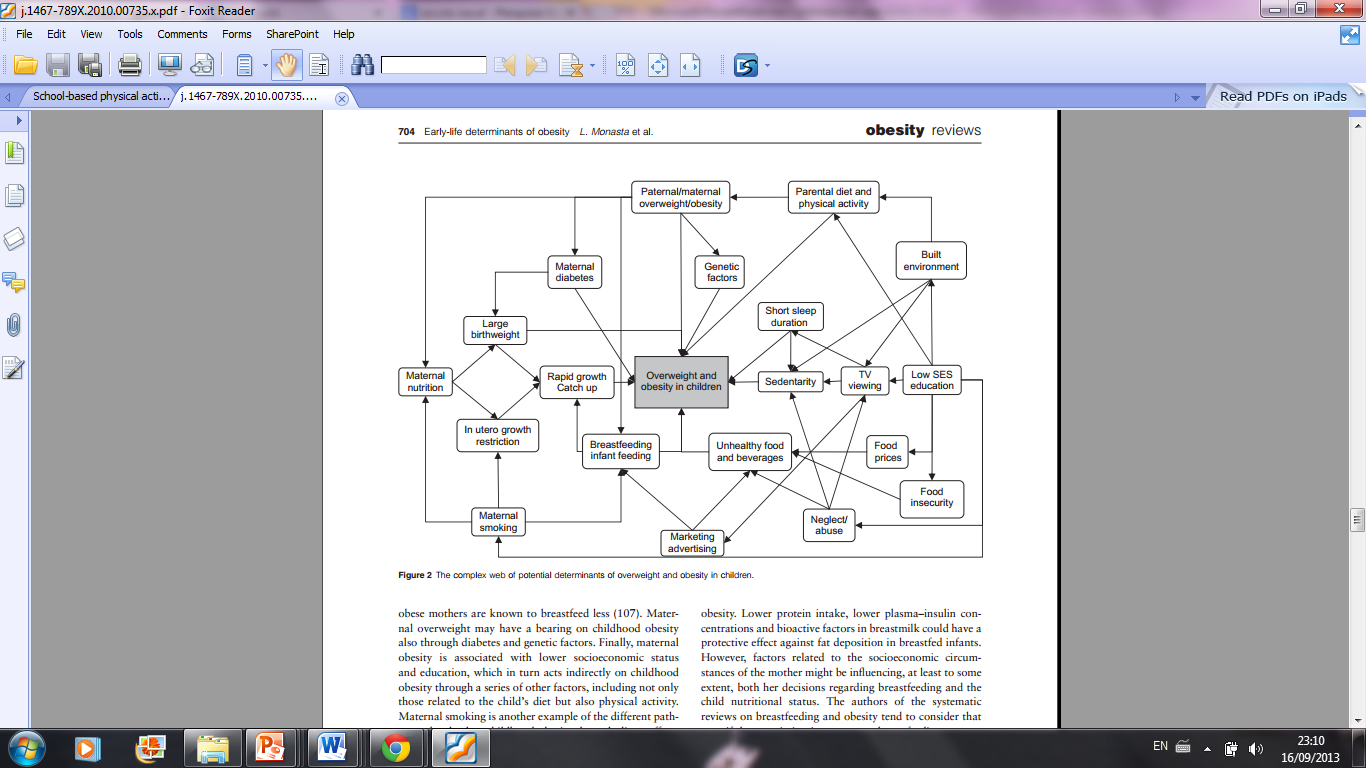


**Figure 1.**  Determinants of obesity among children (Monasta et al., 2010).

Identification of major determinants may help to better understanding obesity relationships and may guide the adoption of public policies.

- 1. **Physical exercise and obesity prevention**

Physical inactivity is one of the main risk factors for the development of several chronic degenerative diseases such as cardiovascular disease, diabetes, osteoporosis and some types of cancer (World Health Organization, 2010). Active lifestyle is associated with less body fat, improved cardiovascular, bone and muscle function, reduced anxiety and depression (Janssen, et al, 2010;. Ravussin, et al., 1988; Zurlo, et al, 1992;. Weinsier, et al, 1998;. Pate, et al, 2013).

Many organizations and scientific societies have published recommendations for the promotion of physical activity and the use of exercise as a strategy to treat obesity, diabetes mellitus and for the prevention of cardiovascular disease (Jakicic, et al., 2001; Bauman, et al., 2005; Warburton et al, 2007). For adults, weekly practice of at least 150 minutes of light or moderate aerobic exercise or 75 minutes of intense aerobic exercises complemented by three weekly sessions of resistance exercise is recommended (World Health Organization, 2010;. Garber, et al, 2011). For children and adolescents it has been recommended daily physical exercise, lasting at least 60 minutes of moderate to vigorous (Tremblay et al., 1994; Landry & Driscoll, 2012).

Hallal et al., (2012) analyzed global levels of physical activity for adults (15 and over) from 122 countries and adolescents (13-15 years) from 105 countries. The results showed that 31.1% of adults worldwide are physically inactive, with proportions ranging from 17% in South Asia to 43% in the Americas and the Eastern Mediterranean. It was also observed that physical inactivity increases with age, is higher among women and higher in high-income countries. For adolescents the percentage not meeting the daily recommendation of 60 minutes of moderate to vigorous physical activity is 80.3%.

Data published by the Ministry of Health on the practice of physical activity among Brazilian adults show that the frequency of sufficient physical activity during leisure time (at least 150 minutes per week of physical activity of mild or moderate intensity or at least 75 minutes a week of physical activity vigorous intensity) is 33.8%, higher among men (41.2%) than among women (27.4%) (Brazil, 2014). Among teenagers, the results of PeNSE 2012 showed that 20.2% of students practiced 60 minutes of physical activity at least five days a week, 27.9% for male students and 13.1% to female (IBGE, 2012).

A large number of studies have evaluated the efficacy of exercise programs in primary and secondary prevention of obesity. These programs have different methodological structure, combining or not exercise with diet (Farias et al, 2009;. Vasquez, et al, 2014.). Other commonly tested approaches include parental involvement, extracurricular activities or recovery of public spaces for physical activity (Ziebarth, et al, 2012;. Branscum & Sharma, 2012; Puder, et al, 2011). .

Early primary prevention school based studies of obesity followed the basis of studies for the prevention of cardiovascular disease. Two large studies entitled Pathways (Lohman et al., 2003) and Planet Health (Gortmaker, et al., 1999) were conducted in US school in the late 90's and both included the practice of physical activities and guidelines on food consumption. They were conducted in populations with high prevalence of obesity, but none of them showed overall reduction in the prevalence of overweight and obesity, similar results were found by other studies such as New Moves (Neumark-Sztainer, et al., 2003).

A systematic review on weight gain prevention programs in adolescents, Kropski et al., (2008) observed that despite not having been found reductions of body weight in 6 of the 14 articles included in the study, positive changes in food intake and physical activity were observed.

Some researchers argue that even if no impact on weight gain, behavioral changes alone are justified considering that the obesity problem is very complex and can not overcome the many factors contributing to the growing epidemic of obesity observed in recent years (Kelly et al., 2008). In addition, most interventions can only address a very small fraction of the factors that generate obesity (Silveira et al., 2011).

On the other hand, some studies have shown that the practice of physical activity can lead to positive results in weight control. The *Kiss* study evaluated the effect of a school based physical activity program on cardiorespiratory fitness and adiposity in 502 children aged 6 to 11 years old in two provinces in Switzerland. The intervention last for nine months, combining increase in weekly frequency of physical education classes from 3 to 5 times a week, 45 minutes per session; 3-5 sessions per day 2-5 minutes of coordination and balance exercises between classes and also 10 minutes of daily home exercise. The results showed increased levels of physical activity and cardiorespiratory fitness and reduced fatness gain compared to the control group (Kriemler, et al., 2010). Similar results were found by the FitKIds study based on 9 months of intervention in physical activity of 220 children 8-9 years randomized to intervention and control group. The intervention consisted in 70 minutes of moderate physical activity, five times a week (Khan et al., 2014).

Overall, reviews on the effect of physical activity on weight control published in recent years have found quite controversial results. In meta-analysis conducted by Harris et al., (2009), school-based physical activity interventions had no impact on BMI of children and adolescents. In line with this study, another systematic review by Dobbins et al., (2013) was also unable to observe impact on BMI of children and adolescents 6-18 years. However, in the systematic review published by Vasconcellos et al., (2014), which analyzed 24 trials investigating the effect of physical activity, alone or in combination with other types of intervention, physical fitness, body composition and other risk factors for cardiovascular disease were improved in obese adolescents.

In an attempt to understand the conflicting findings in the literature, Metcalf et al., (2012), in a systematic review of preventive studies based on physical activity, argues that the studies have very little effect on increasing physical activity, in the order of 4 minutes of walking or jogging a day, which could explain, at least in part, the lack of effect of these interventions in reducing or maintaining the BMI in children. Thus, the importance of exercise in the prevention of obesity as well as in the process of weight reduction in adolescents, is still not well understood.

This inconsistency in results can be partly explained by the short period of interventions, large methodological differences in the assessment of body composition and also due to the different structures of the exercise protocols used in interventions (type of employee exercise duration and intensity of the sessions), and the failure of interventions in an attempt to increase the overall time of physical exercise.

- 1. **Effects of exercise in the energy expenditure and quantity of daily physical activity**

The complexity of body weight regulation is a major challenge for understanding the etiology, treatment and prevention of obesity. Although intensively studied, there are still many controversies, including its relationship with exercise and total energy expenditure (Roth, et al., 2004).

Van der Heijden et al., (2010) tested the hypothesis of an increase in the 24 hours energy expenditure and fat oxidation after moderate training of 43 lean and obese adolescents. They participated in an aerobic exercise program four times a week with a heart rate above 70% of maximum oxygen consumption (VO2 max). After 12 weeks of training, the exercise did not increase the total energy expenditure, whereas fat oxidation increased in lean adolescents, there was no change in the obese. It is noteworthy in this study the classification as moderate intensity for a heart rate above 70% of VO2 max. According to the literature, moderate intensity is classified into the range of 46% to 63% of VO2 max and vigorous range 64% to 90% of VO2 max (Physical Activity Advisory Committee Guidelines, 2008).

A compensatory effect that physical exercise can promote in subsequent physical activities has been discussed by Epstein & Wing (1980) based on a meta-analysis of the effect of aerobic exercise on weight loss and body fat. Authors observed that the subjects who undergo exercise sessions lost less weight than expected. Two possibilities for explaining this event: the first was that exercise stimulated the appetite, with increased caloric intake; the second hypothesis is that individuals who exercised showed the level of physical activity modified the rest of the day, ie, moved up less due to a supposed "fatigue" induced by exercise with a reduction of total energy expenditure. From there, in the 90s, early studies tested the compensatory effect caused by exercise. Meijer et al., (1991) recruited 32 adults, male and female, for a 20 weeks training program for a half marathon. The study results suggest that exercise training increased the amount of physical activity performed, measured with accelerometers, in the twentieth week in men and women (62% and 63%, respectively). Therefore, a high volume of training associated with a vigorous intensity can increase the amount of daily physical activity in adults, not supporting the hypothesis offsetting effect.

Later, Goran & Poehlman (1992) evaluated the effect of aerobic exercise on total energy expenditure in healthy elderly. The exercises were performed in exercise bike, three times a week for 8 weeks. After training, the total energy expenditure assessed by doubly labeled water method, did not change compared to the pre-training period. Even with the increase in resting energy expenditure and energy expenditure promoted by exercise sessions, subjects became less active during the day, a decrease in energy expenditure promoted by spontaneous physical activity. The authors argue that the results may be related to vigorous intensity that individuals were exposed in recent weeks of training, results similar to those found in other studies that evaluated the effect of moderate physical exercise sessions (Meijer, et al., 1999; Meijer et al., 2000).

Blaak et al. (1991) were the first to investigate the hypothesis of compensatory effect caused by exercise in children. In this study, ten obese children 10 to 11 years of age participated in an aerobic training program for 4 weeks. The subjects performed cycle ergometer exercise, five times a week for 60 minutes at moderate intensity. The study results indicate that adding one hour of exercise a day provides a significant increase in daily energy expenditure in obese boys, but changes in the practice of spontaneous physical activity were observed.

In an attempt to explain this offsetting effect phenomenon, Rowland (1998) first described the hypothesis of "Activitystat", defining as a homeostatic mechanism where a biological control center would be responsible for the control of physical activity according to a set point of energy expenditure. It is a mechanism that allows the continued stability in a dynamic system through a negative feedback process. When an imbalance occurs, regulatory systems become active to restore baseline (Guyton and Hall, 2006). According to this theory, the increase or decrease in physical activity levels at any given time would be offset by changing these levels at another time, in defense of an individual set point. The type, duration and intensity of exercise are important components to be considered (Gomersall, et al., 2013).

Dale et al., (2000) were the first to publish data with specific reference to Activitystat showing no compensation between physical activity in the period at school and out of school. Four years later, Metcalf et al., (2004), using accelerometers, compared the amount of physical activity performed in the week between those who would walk to school and those who were taken by car and showed no difference in the total amount of weekly physical activity. On the other hand, Lynch et al., (2009) comparing among adults workout days with days without exercise observed no difference between the amount of physical activity performed on days with and without exercise excluding the workout and the authors conclude that physical exercise does not promote reduction in spontaneous physical activity. Therefore, although observed in elderly (Poehlman & Goran, 1992; Meijer et al., 1999;. Meijer, et al, 2000) the compensation was not observed in young adults.

Among children Wilkin et al., (2006) demonstrated that the total amount of physical activity performed was similar among those children who attended schools with a high volume of physical education classes compared to those with few classes. Corroborating these findings, Frémeaux et al., (2011) evaluated whether a higher volume of physical activity during school hours could be offset by lower volume of physical activity in the period out of school showing that increased school physical activity induces a reduction in spontaneous physical activity outside of school, without significant difference in the amount of weekly physical activity among schools. However, other studies that assessed the effects of different amounts of physical activity on subsequent activities found different results, such as the study by Wickel & Eisenmann, (2007) with sports practice among 36 children, the study by Baggett et al., (2010), analyzing data from 6916 girls and also the study by Goodman, et al., 2011).

The greater difference in the methods employed can be one reason for the differences found in the studies since exercise programs are structured taking into account factors such as: the type of exercise (aerobic, resistance or flexibility), the duration, the weekly frequency and intensity of the activity (light, moderate or vigorous) and also the various outcome measures.

Regarding intensity of the exercise, it is believed that mild exercise would be the most appropriate to be prescribed for obese individuals, mainly due to the higher proportion of oxidized lipids in this intensity zone (Achten, et al. 2002). However, total energy expenditure (TEE) should be the focus when the primary goal is weight reduction. The ideal area intensity of exercise to optimize the GET and consequent reduction of fat coporal is still under discussion.

In a cross-sectional study, Tremblay et al., (1990) evaluated the effect of different intensities of physical activity on body fat of 2623 adults, of both sexes, who participated in the Canadian physical fitness survey in 1981 (Stephens, et al., 1986). Physical activity was assessed through questionnaires and body fat by skinfold method. The authors concluded that any vigorous physical activity on a regular basis for more than 6 months reduce body fat and waist-hip ratio, indicating that increasing the intensity of exercise may promote a negative energy balance. Four years later, authors assessed the impact of the intensity of exercise on weight and body fat through exercise program cycle ergometer for 15 to 20 weeks, among 27 healthy adults of both sexes. Results showed that body weight did not changed, but subcutaneous fat was significantly lower in the group of high intensity exercise (Tremblay et al., 1994). In a study with similar method Trapp et al., (2008) compared the effects of intermittent high-intensity exercise with continuous moderate exercise performed for 15 weeks in 45 healthy women and only the high-intensity group showed a significant reduction of total body weight, fat weight and plasma insulin levels.

On the other hand, there are studies showing no difference due to exercise intensity in weight or body fat such as the study by Jakicic et al, (2003) among 201 overweight and sedentary individuals. After 12 months of training, it was observed reduction in body weight and improved cardiorespiratory fitness in all groups despite of the intensity of the exercise.

In another study, Duncan et al., (2005) compared the effects of the hike in different intensities and weekly frequency in cardiorespiratory fitness and lipid profiles in 492 healthy adults. The experimental protocol contemplated four different conditions: moderate / low frequency, moderate intensity / high frequency, high intensity / low frequency and high intensity / high frequency. The training area was determined at 45% to 55% and 65% to 75% of the heart rate reserve for moderate and strong intensities, respectively. Participants in the low frequency weekly group were instructed to walk 3 to 4 times a week while the high-frequency group of 5 to 7 days per week. After 24 months, none of the groups showed statistically significant change in body weight. Also, Tjonna et al., (2008) examined the effects of exercise training versus intense in variables associated with cardiovascular function in 32 patients with metabolic syndrome and concluded that the two exercise programs were equally effective in reducing blood pressure, weight and body fat.

Wang et al. (2011) randomized 36 obese postmenopausal women in two groups. The first group was asked to perform treadmill walking at a moderate intensity (45% - 50% of maximal oxygen consumption) while the second group at a vigorous intensity (70% - 75% of maximum oxygen consumption). After five months energy expenditure among the women in the group who performed moderate activity was higher on days when they performed the exercise (577.7 ± 219.7 kcal.d-1) compared to the days when not performed ( 450.7 ± 140.5 kcal.d-1), however, the overall difference was much lower than the one spent on the sessions (127.0 ± 188.1 kcal.d-1). In women who performed vigorous exercise, energy expenditure on days with exercise (450.6 ± 153.6 kcal.d-1) was lower compared to days without exercise (519.2 ± 127.4 kcal.d-1) . The authors conclude that there is a compensatory effect caused by exercise that occurs primarily with high-intensity activities.

Kriemler et al., (1999) evaluated the impact of activity in stationary bike in energy expenditure and amount of physical activity practiced by 14 obese adolescents during different intensity sessions. The first was vigorous intensity with four sets of 10 minutes with 5 minutes apart in (heart rate of 150-160); the second, they were asked to perform two sets of 15 minutes with 5 minutes interval of moderate intensity (heart rate 130-140). After each exercise session, energy expenditure and the amount of physical activity was increased in the moderate intensity group, whereas vigorous intensity leads to reduced subsequent physical activities.

In a recent study comparing the effect of different intensities of exercise training on the daily energy expenditure in normal weight and obese subjects, Thivel et al., (2013) concluded that while vigorous exercise can contribute to an improvement body composition, physical fitness and metabolic profile (Boutcher, 2011), with obese adolescents showing a compensatory response to a high-intensity workout (above 70% of VO2 max) decreasing the energy expenditure of subsequent physical activities, but not adolescents with normal weight. Therefore, studies that evaluate the effects of different exercise intensities on daily energy expenditure and their compensatory effects on spontaneous physical activity are still controversial.

1. **Rationale**

Exercise has long been regarded as an important strategy in many obesity-reduction programs, however most studies have showed that practice of physical exercise has not contributed significantly to weight or body fat reduction (Swift, et al., 2014). This inconsistency in results can be partly explained by the short period of interventions, differences in methods employed in the evaluation and the outcomes measured. Also, type of exercise, frequency, duration and intensity of exercise sessions may interact differently with each outcome studied.

In Brazil and other countries guidelines on physical activity have recommended for children and adolescents daily physical exercise, lasting at least 60 minutes of moderate to vigorous for health and prevention of obesity and other diseases (Landry & Driscoll, 2012). However, specifically for weight reduction, exercise intensity is still a point of discussion in the literature.

Strenuous exercise compared with moderate (Tremblay, et al., 1994; Trapp, et al, 2008) appears to show greater weight loss in some but not all (Jakicic et al. 2003; Tjonna, et al, 2008) studies. Therefore, most people believe that moderate exercise and reduce sedentary activities should be encouraged as a better alternative to increase the daily energy expenditure and to reduce body weight (Westerterp & Plasqui, 2004), mainly because of obese individuals feel more comfort and confidence with the practice of moderate exercise (Piana, et al., 2013). However, few studies have also evaluated the effect of different intensities of exercise on daily energy expenditure in children with overweight.

1. **Objective**

To evaluate the effect of two intensities of exercise on the subsequent spontaneous physical activity energy expenditure among overweight adolescent.

1. **Method**
   1. **Study design**

This is an experimental crossover design, where all selected subjects will undergo three experimental conditions.

All adolescentes enro lled in the 6th and 7th grade in a public school in Niterói, Rio de Janeiro were invited to undergo anthropometric measurements to determine de body mass index (BMI). Only those adolescents classified as overweight according to World Health Organization parameters for age and sex (de Onis, et al., 2007) were included in the study. In order to minimize the risk of stigmatization, eutrophic adolescents will be invite to participate in exercise sessions, with the amount corresponding to 20% of the sample size.

The adolescents will be assessed in five visits. During the first visit, anthropometric measures will be collected. During the second visit, the adolescents will undergo a maximum field test to evaluate aerobic capacity (Leger, et al., 1988). At the end of the test, the maximum heart rate will be determined and the heart rate zone for each experimental session will be calculated. The third and fourth visits will be experimental sessions with different training intensities and the fifth session will be the control session (no exercise), interspersed by a period of one week.(**figure 2**).


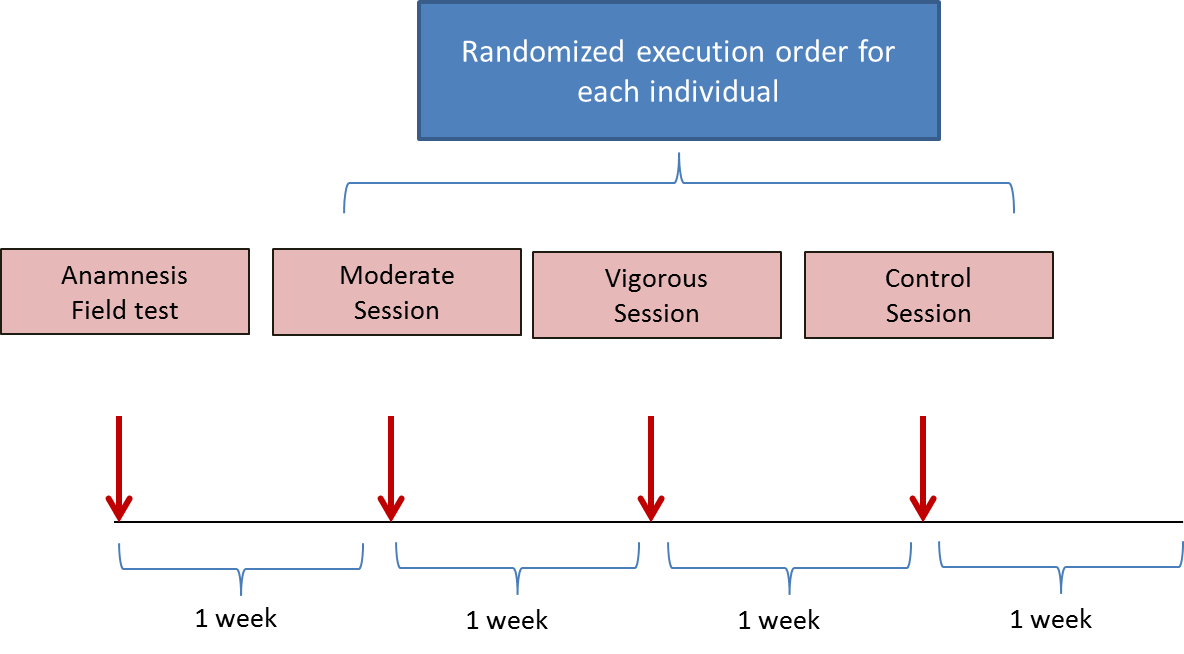


**Figure 2.** Scheme of the experimental sessions

The experimental sessions will be structured in two phases: warm-up, training, cool-down. The exercise protocol is different between the experimental sessions (moderate and vigorous) only in the training phase, with similar warm-up and cool-down phases in both sessions. During the warm-up, subjects will be instructed to start walking at a low-intensity, and then increase their pace until they reached values close to 64% of maximum heart rate. In the cool-down phase, adolescents will be instructed to gradually reduce their pace in order to reach heart rate values close to those found at rest. (**figure 3**).


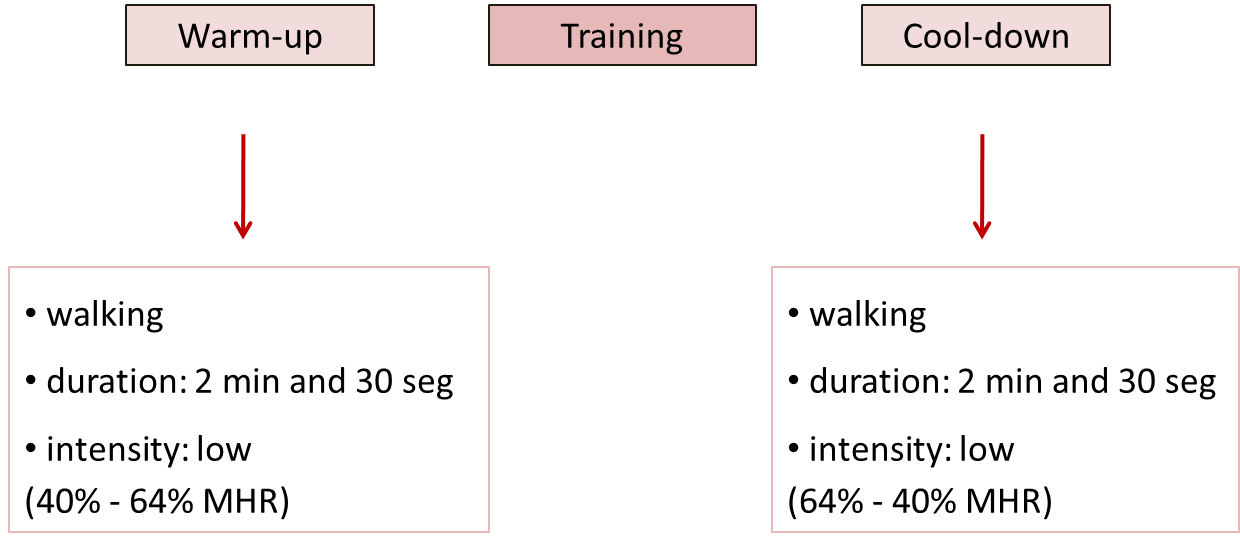


**Figure 3.** Warm-up and cool-down characteristics

The main part of the moderate session (MS) consist of 4 sets of 10 minutes walking at moderate intensity (64% to 76% of maximum heart rate), interspersed with 5 minutes of light walking (below 64% of maximum heart rate) for recovery between sets. (**figure 4**).


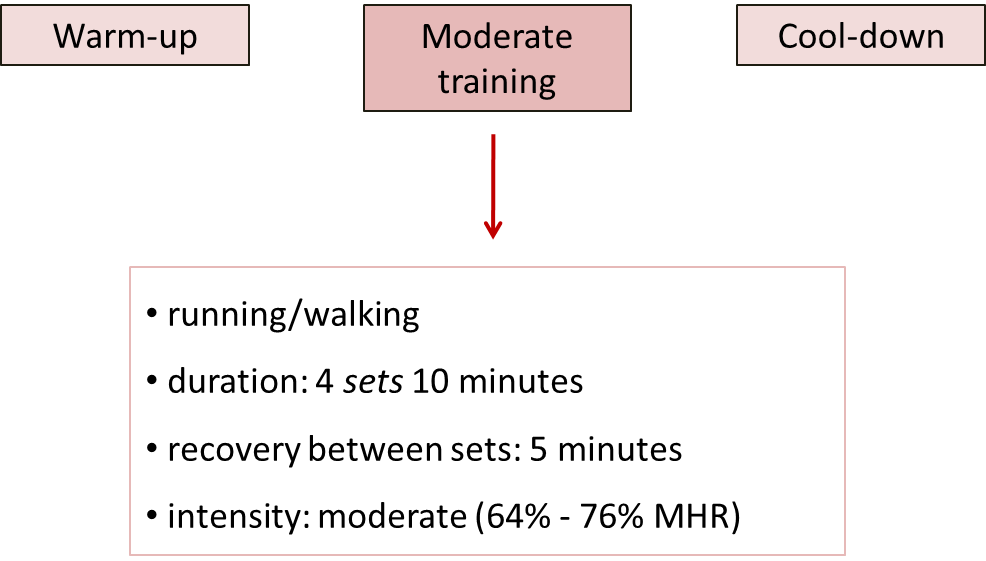


**Figure 4.** Main part of the moderate session

The main part of vigorous session (VS) consist on 4 sets of 10 minutes running at vigorous intensity (77% to 95% of maximum heart rate), interspersed with 5 minutes of light walking (below 64% of maximum heart rate) for the recovery between sets. (**figure 5**).


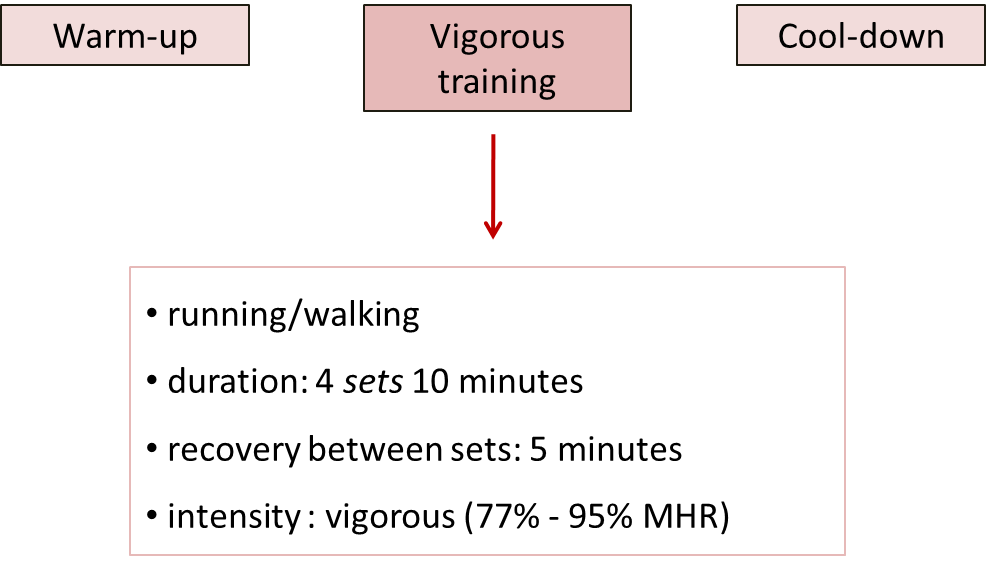


**Figure 5.** Main part of the vigorous session

During the two experimental sessions, the adolescents will be supervised by a trained exercise physiologist to ensure that they will perform the intervention protocol properly. The target heart rate will be monitored using heart rate monitors. One week was defined as a washout period (**figure 2**).

During the exercise sessions and for a period of six days, energy expenditure and the amount of physical activity will be evaluated through accelerometers. It consists of a small device that is placed at the hip region and should be removed only during bathing or during the practice of aquatic activities. The use of this device is considered safe, however, for an extended period, may cause discomfort. The adolescents will be informed not to perform physical exercises in the three days before each session, and not drinking alcohol, coffee or any other stimulant in the 24 hours prior to each session.

- 1. **Sample size**

Sample size was calculated based on a mean difference in daily energy expenditure between sessions of 110 kcal (Wang, et al., 2006) with a coefficient of variation equal to 1 (standard deviation of 110 kcal) (van Belle, 2008). The sample size required for the study, with an α of 0.05 and β of 0.10 and estimating a refusal rate of 20%, was 27 children (Julious, 2009).

- 1. **Measurement procedures**
     1. **Anthropometric measurements**

Body weight will be measured using a portable electronic scale (Tanita BC -558 Japan) with a capacity of 150 kg and precision of 50g, while wearing light clothes and no shoes. Height will be measured using a portable stadiometer (Alturexata, Brazil) with an amplitude of 200 cm and variation of 0.1 cm. Participants will be instructed to remain without shoes, feet together and keeping the head in the Frankfurt plane (Madsen, et al., 2008). Two measurements will be made and considered the mean values ​​for analysis. The classification of nutritional status will be based on BMI (kg/m^2^) cutoff points recommended by the World Health Organization (de Onis, et al., 2007).

- - 1. **Shuttle run test**

Cardiorespiratory fitness will be assessed by a maximum field test, specific to the age of the study group. The shuttle run, is a valid test (Leger et al., 1988) and widely used in studies with adolescents worldwide (Jimenez-Pavon et al ., 2013; veses, et al, 2014). The purpose of this test is to estimate the maximum oxygen consumption. It is a progressive, maximum and indirect test. Participants are placed at the starting line and begin the test with the first beep. They must arrive at the marked location, bypassing the line, before sounding the next beep. The direction changes must be made to stop and start the other way, avoiding curvilinear paths. Every minute, the time between beeps will decrease, which mean an increase in the speed. The test will be completed with the participant's withdrawal, or when it fails to reach the scored line two consecutive times. A doctor will be present during the test for possible complications. Each participant will use a heart rate monitor during the test and the maximum heart rate will be recorded. Thus, the intensity of each exercise session (moderate and vigorous) will be calculated, individually, according to the percentage of the maximum frequency established for moderate and vigorous intensity (Physical Activity Guidelines Advisory Committee, 2008).

- - 1. **Energy expenditure measurements**

The physical activity energy expenditure during one week will be assessed through Actigraph accelerometers, wGT3X-BT model. It is a portable, lightweight and non-invasive device, providing an objective measurement of physical activity. The accelerometer will be placed on the day before each experimental session, at the same time and removed after one week.

This device is often used in epidemiological studies to evaluate the recommendations of physical activity for children and adolescents, as well as providing an estimate of energy expenditure derived from the activities. In order to proceed with the evaluations, threshold values ​​were developed to fractionate the intensity into categories: sedentary, light, moderate and intense.

The border points that will be used for each intensity range have been proposed by Evenson (2008), which below 100 counts per minute, physical activity is classified as sedentary between, 100 and 2296 counts per minute as light activity, between 2296 and 4012 as moderate and above 4012 vigorous physical activity. These points have shown greater precision to classify physical activity intensity than other points established in other studies (Trost, et al., 2011).

The sampling period (epoch) in this study is 5 seconds to capture the possibility of minor variations in movements performed by adolescents (Trost, et al., 2005). Days with less than 600 minutes of recorded data or more than 60 minutes counts of consecutive zeros, will be excluded from the analysis (Choi et al., 2011).

Adolescents will be instructed not to take out the device during that time, except for the period of the bath and while practicing water activities.

- 1. **Data analysis**

The descriptive analyses will calculate the mean and standard deviations for continuous and percentages for categorical variables. Differences in daily and accumulated energy expenditure between exercise sessions will be performed using linear mixed models, which takes into account the correlations between repeated measures over time.

The analysis will be performed in SAS 9.3 software (Statistical Analysis System, USA) and statistical significance set at p<0.05 for all analyses.

•

•

•

•

1. **References**

Achten J, Gleeson M, Jeukendrup A. Determination of the exercise intensity that elicits maximal fat oxidation. Med Sci Sports Exerc 2002; 34: 92-97.

American College of Sports Medicine. ACSM’ Guidelines for exercise testing and prescription. 8 ed. Lippincott Willians & Wilkins, Philadelphia, 2009.
